# Plastome phylogenomics reveals an early Pliocene North- and Central America colonization by long-distance dispersal from South America of a highly diverse bromeliad lineage

**DOI:** 10.3389/fpls.2023.1205511

**Published:** 2023-06-23

**Authors:** Sandra I. Vera-Paz, Carolina Granados Mendoza, Daniel D. Díaz Contreras Díaz, Matthias Jost, Gerardo A. Salazar, Andrés J. Rossado, Claudia A. Montes-Azcué, Rebeca Hernández-Gutiérrez, Susana Magallón, Luis A. Sánchez-González, Eric J. Gouda, Lidia I. Cabrera, Ivón M. Ramírez-Morillo, María Flores-Cruz, Xochitl Granados-Aguilar, Ana L. Martínez-García, Claudia T. Hornung-Leoni, Michael H.J. Barfuss, Stefan Wanke

**Affiliations:** ^1^ Departamento de Botánica, Instituto de Biología, Universidad Nacional Autónoma de México, Mexico City, Mexico; ^2^ Posgrado en Ciencias Biológicas, Universidad Nacional Autónoma de México, Mexico City, Mexico; ^3^ Institut für Botanik, Technische Universität Dresden, Dresden, Germany; ^4^ Laboratorio de Sistemática de Plantas Vasculares, Facultad de Ciencias, Universidad de la República, Montevideo, Uruguay; ^5^ Departament of Evolution, Ecology, and Organismal Biology, University of California, Riverside, CA, United States; ^6^ Museo de Zoología “Alfonso L. Herrera”, Departamento de Biología Evolutiva, Facultad de Ciencias, Universidad Nacional Autónoma de México, Mexico City, Mexico; ^7^ Botanical Garden, Utrecht University, Utrecht, Netherlands; ^8^ Herbario CICY, Centro de Investigación Científica de Yucatán, A.C. (CICY), Yucatán, Mexico; ^9^ Departamento El Hombre y su Ambiente, División de Ciencias Biológicas y de la Salud, Universidad Autónoma Metropolitana, Unidad Xochimilco, Mexico City, Mexico; ^10^ Departamento de Ecología Evolutiva, Instituto de Ecología, Universidad Nacional Autónoma de México, Mexico City, Mexico; ^11^ Centro de Investigaciones Biológicas, Herbario HGOM, Instituto de Ciencias Básicas e Ingeniería, Universidad Autónoma del Estado de Hidalgo, Hidalgo, Mexico; ^12^ Departament of Botany and Biodiversity Research, University of Vienna, Vienna, Austria

**Keywords:** rapid diversification, secondary calibration, ancestral area estimation, Hyb-Seq, phylogenomic dating

## Abstract

Understanding the spatial and temporal frameworks of species diversification is fundamental in evolutionary biology. Assessing the geographic origin and dispersal history of highly diverse lineages of rapid diversification can be hindered by the lack of appropriately sampled, resolved, and strongly supported phylogenetic contexts. The use of currently available cost-efficient sequencing strategies allows for the generation of a substantial amount of sequence data for dense taxonomic samplings, which together with well-curated geographic information and biogeographic models allow us to formally test the mode and tempo of dispersal events occurring in quick succession. Here, we assess the spatial and temporal frameworks for the origin and dispersal history of the expanded clade K, a highly diverse *Tillandsia* subgenus *Tillandsia* (Bromeliaceae, Poales) lineage hypothesized to have undergone a rapid radiation across the Neotropics. We assembled full plastomes from Hyb-Seq data for a dense taxon sampling of the expanded clade K plus a careful selection of outgroup species and used them to estimate a time- calibrated phylogenetic framework. This dated phylogenetic hypothesis was then used to perform biogeographic model tests and ancestral area reconstructions based on a comprehensive compilation of geographic information. The expanded clade K colonized North and Central America, specifically the Mexican transition zone and the Mesoamerican dominion, by long-distance dispersal from South America at least 4.86 Mya, when most of the Mexican highlands were already formed. Several dispersal events occurred subsequently northward to the southern Nearctic region, eastward to the Caribbean, and southward to the Pacific dominion during the last 2.8 Mya, a period characterized by pronounced climate fluctuations, derived from glacial–interglacial climate oscillations, and substantial volcanic activity, mainly in the Trans-Mexican Volcanic Belt. Our taxon sampling design allowed us to calibrate for the first time several nodes, not only within the expanded clade K focal group but also in other Tillandsioideae lineages. We expect that this dated phylogenetic framework will facilitate future macroevolutionary studies and provide reference age estimates to perform secondary calibrations for other Tillandsioideae lineages.

## Introduction

Modeling when and where diversification of a lineage took place requires sound and appropriately sampled time-calibrated phylogenetic hypotheses, along with information about the geographic distribution of the species of interest and testing of explicit biogeographic models. Species occurrence records can nowadays be readily accessed through several public databases, such as the Global Biodiversity Information Facility (https://www.gbif.org/es/), which, combined with automated curation strategies (e.g., CoordinateCleaner; Zizka et al., 2020), expert knowledge, and newly collected field information, represent a valuable and reliable source of geographic information. Biogeographic models using *a priori*-defined discrete areas are particularly useful for the reconstruction of biogeographic patterns of lineages above the species level, where continuous biogeographic models can be obscured by the more likely movement of organism within areas (Ronquist and Sanmartín, 2011). Generating molecular phylogenetic hypotheses for species-rich lineages with rapid diversification still poses serious challenges associated with the scarcity of informative data for divergences occurring in quick succession (short internodes, e.g., Leaché and Rannala, 2011; Wickett et al., 2014; Prum et al., 2015; Wanke et al., 2017), as well as with the effort required to generate sequence data for dense taxon samplings. Hyb-Seq is a cost-effective sequencing strategy that allows the simultaneous generation of nuclear, biparentally inherited loci through target enrichment, along with uniparentally inherited organellar (i.e., plastome and mitochondrial) data *via* genome skimming (Weitemier et al., 2014; Dodsworth et al., 2019). When properly designed, Hyb-Seq strategies allow for the recovery of significant amounts of plastid data (Granados Mendoza et al., 2020) or even complete organellar genomes (Vera-Paz et al., 2022), which represent a valuable source of data for phylogenetic inference. Plastome-scale data, and particularly non-coding regions, have shown to substantially improve phylogenetic resolution and statistical support compared with approaches incorporating only or a few coding regions (Givnish et al., 2018). In addition to the amount of sequence data required to achieve phylogenetic resolution for rapidly evolving lineages, generating dated phylogenetic frameworks requires sampling key nodes where fossil-based or secondary calibrations can be established, which often requires sampling much deeper divergences than those of the group of interest (e.g., Givnish et al., 2004; Givnish et al., 2011).

Bromeliads are known to have undergone one of the most remarkable plant adaptive radiations in the Neotropics (Givnish et al., 2007; Givnish et al., 2011). The phylogenetic position of Bromeliaceae relative to the other families of the order Poales is still uncertain. A recent monocot-wide plastome phylogenetic study placed Bromeliaceae as sister to all other Poales families, albeit with low statistical support (Givnish et al., 2018), which, for instance, differs from the highly supported hypothesis of Bromeliaceae sister of Typhaceae recovered in the multilocus nuclear analysis of the Tree of Life Explorer (https://treeoflife.kew.org/tree-of-life). A single fossil, *Karatophyllum bromelioides* L.D. Gómez, has unambiguously been assigned to Bromeliaceae (Gómez, 1972; Kessous et al., 2021); however, its use in phylogenetic dating studies has been hampered by the uncertainty around its collection locality and, therefore, its age (Baresch et al., 2011). Regardless of the phylogenetic position of Bromeliaceae within Poales, phylogenetic dating studies using secondary calibrations coincide in that after diverging from its sister lineage, this family underwent an evolutionary stasis of around 81–100 My before diversifying into its modern lineages (Givnish et al., 2011; Givnish et al., 2018; Ramírez-Barahona et al., 2020). The geographic patterns of Bromeliaceae diversification have been addressed at the subfamily level in a key study by Givnish and collaborators (2011). However, the study of the evolution in time and space of some highly diverse lineages of the bromeliad genus *Tillandsia* (subfamily Tillandsioideae, tribe Tillandsieae; Barfuss et al., 2016) has been hindered by the lack of resolved and strongly supported phylogenetic frameworks. Molecular phylogenetic studies in this genus have mostly used Sanger sequenced plastid, nuclear-ribosomal, or nuclear low copy markers (Terry et al., 1997; Barfuss et al., 2005; Granados Mendoza, 2008; Chew et al., 2010; Sidoti, 2015; Barfuss et al., 2016; Castello et al., 2016; Pinzón et al., 2016; Granados Mendoza et al., 2017; Montes-Azcué, 2020; Ancona et al., 2022; Donadío et al., 2022). However, studies applying next- generation sequencing strategies to *Tillandsia* representatives significantly increased in recent years, (Loiseau et al., 2019; Loiseau et al., 2021; Möbus et al., 2021; Vera-Paz et al., 2022; Yardeni et al., 2022; Groot Crego et al., 2023), opening exciting possibilities for addressing fundamental macroevolutionary and biogeographic questions in this genus.

**Figure 1:**
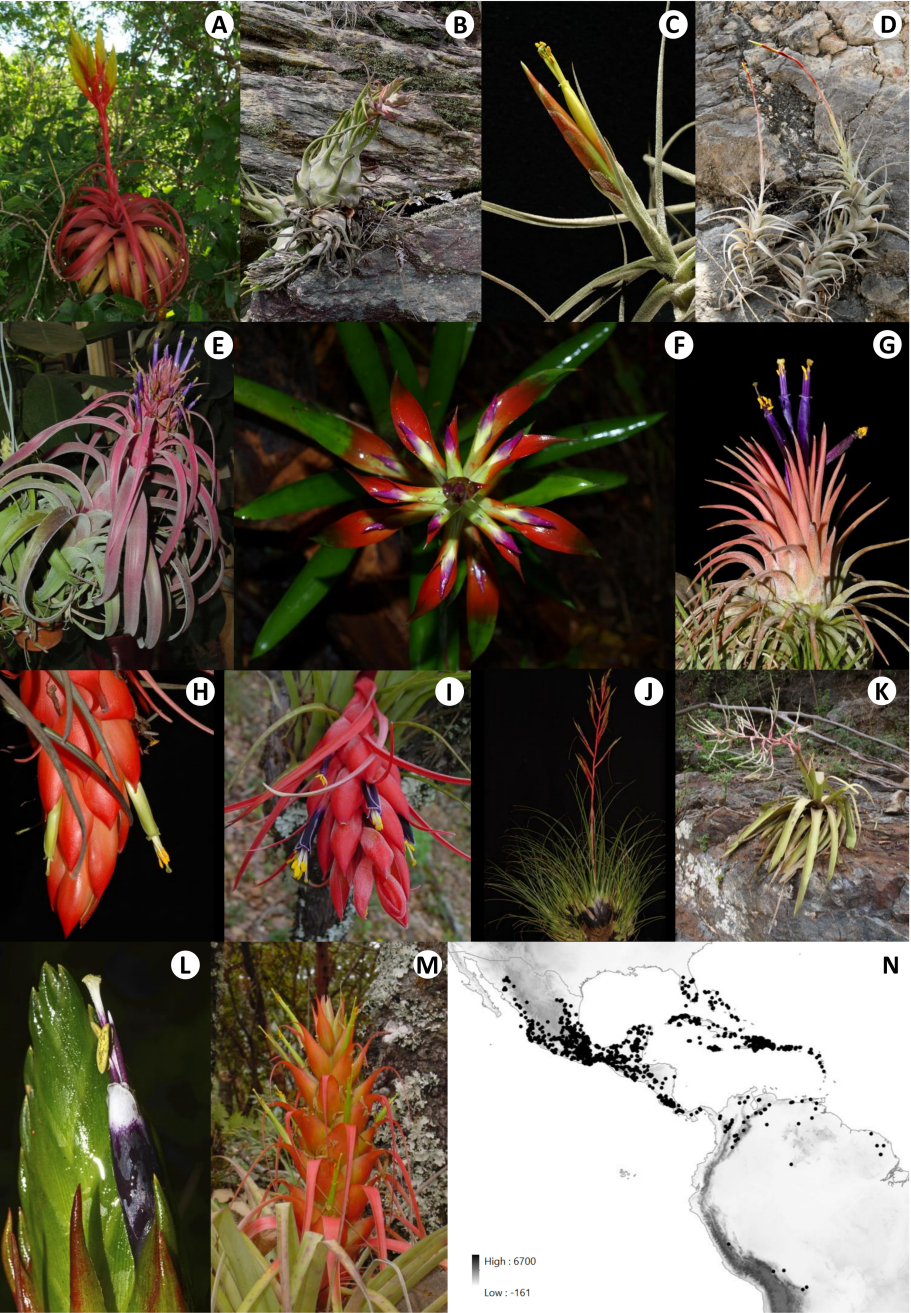
Morphological diversity and geographic distribution of the focal group expanded clade K. Representatives of the expanded clade K: **(A)** *Tillandsia rothii* (Granados Mendoza, 1058), **(B)** *T. seleriana* (Ramírez-Morillo, 971), **(C)** *T. schiedeana* (Granados Mendoza, 297), **(D)** *T. albida* (Hornung-Leoni, s/n), **(E)** *T. jaguactalensis* (Ramírez-Morillo, s/n), **(F)** *T. leiboldiana* (Granados Mendoza, 832), **(G)** *T. ionantha* (Salazar, s/n), **(H)** *T. quaquaflorifera* (Granados Mendoza, 836), **(I)** *T. oaxacana* (Ramírez-Morillo, 1396), **(J)** *T. kirchhoffiana* (Granados Mendoza, 445), **(K)** *T. joel-mandimboensis* (Flores-Cruz, 2286), **(L)** *T. punctulata* (Salazar, s/n), and **(M)** *T. carlos-hankii* (Ramírez-Morillo, 1395). **(N)** Geographic distribution of the expanded clade K. Source of map: Modified from https://www.worldclim.org/data/worldclim21.html (elev 30s, Fick and Hijmans, 2017) and http://tapiquen-sig.jimdo.com (Carlos Efraín Porto Tapiquén. Orogénesis Soluciones Geográficas. Porlamar, Venezuela 2015. Based on layers from the Enviromental Systems Research Institute - ESRI). Photographers: Granados Mendoza **(A, F, H, J, K)**, Ramírez-Morillo **(B, E, I, M)**, Loyola **(C)**, Hornung-Leoni **(D)**, and Salazar **(G, L)**.

*Tillandsia*, as circumscribed by Barfuss et al. (2016), is the most diverse bromeliad genus, with ca. 780 species distributed from the southern USA to Argentina and Chile (Zizka et al., 2020; Gouda et al., continuously updated). Seven subgenera are currently recognized for this genus, of which *T.* subg. *Tillandsia* is the most diverse (> 270 spp.; Barfuss et al., 2016). Barfuss et al. (2005) proposed that *T.* subg. *Tillandsia*, therein equivalent to the later referred clade K, migrated from the Andes northward to North and Central America and identified Mexico and Central America as its centers of diversity. Möbus et al. (2021) recovered stem and crown ages of ca. 7.4 and 6.6 Mya, respectively, for this subgenus, suggesting a recent and rapid diversification from the mid-Neogene and onward. According to Barfuss et al. (2016), three main lineages in successive sister relationship compose *T*. subg. *Tillandsia*, the first of them including *T. hildae*, *T. ferreyrae*, *T. heliconioides*, *T. malzinei*, and *T. paniculata* from Mexico, Central America, the Caribbean, and South America (hereafter referred to as “*T. paniculata* clade”). The second includes *T. propagulifera*, *T. spiraliflora*, *T. ecarinata*, *T. secunda*, and *T. adpressiflora* from South America (hereafter referred to as “*T. secunda* clade”). The third lineage, named “clade K” and focus of the present study, was first identified by Barfuss et al. (2005), and at that time it was known to include 11 species from Mexico, Central America, the Caribbean, and South America. Subsequent studies have consistently recovered clade K with strong statistical support. Despite the limited resolution of their phylogenetic context, resulting from the use of a single plastid region (*mat*K*-trn*K), Granados Mendoza et al. (2017) uncovered that this lineage is considerably more diverse (82 spp.), renamed it as “expanded clade K,” and named its two main internal clades as K.1 and K.2. The species of the expanded clade K are mainly distributed in North and Central America, with some species also in northern South America and the Caribbean (Granados Mendoza et al., 2017; **Figure 1**). This clade includes species with both crassulacean acid metabolism (CAM) and C3 photosynthesis (Crayn et al., 2015; De La Harpe et al., 2020; Groot Crego et al., 2023), and its species inhabit a wide range of mesic to xeric habitats, from sea level to 3,300 m elevation (Espejo-Serna et al., 2004; Gouda et al., continuously updated; **Figure 1**). To our knowledge, no study has provided a resolved and strongly supported dated phylogeny representing the various lineages composing the expanded clade K, therefore hindering further macroevolutionary and biogeographic studies in this highly diverse lineage. A series of molecular and morphological studies have focused on exploring phylogenetic relationships and/or testing species delimitations of specific species complexes and groups within the expanded clade K (Granados Mendoza, 2008; Chew et al., 2010; Sidoti, 2015; Pinzón et al., 2016; Ancona et al., 2022; Martínez-García et al., 2022), many of them identified by Gardner (1986), albeit with a limited sampling of other lineages of the focal group.

The present study aims at reconstructing the spatial and temporal frameworks of the origin and diversification of the expanded clade K. To achieve this, we generated high-quality plastome assemblies from Hyb-Seq data for a dense taxon sampling of our focal group plus a careful selection of outgroup species. We then used this substantial amount of sequence data to estimate a time-calibrated phylogeny and perform ancestral area reconstructions based on information from a well-curated geographic occurrence database.

## Material and methods

### Taxon sampling

Full plastomes of a total of 200 species were analyzed, of which 162 were newly sequenced and assembled for this study and 38, generated by Guisinger et al. (1 sp.; 2010) and Vera-Paz et al. (37 spp.; 2022), were downloaded from GenBank (**Supplementary Material 1**). The sampled species were selected to construct a phylogenetic context with key nodes represented for 1) performing secondary calibrations, i.e., stem and crown nodes of Bromeliaceae and the crown node of Tillandsioideae, and 2) reconstructing the geographic origin and dispersal history of the expanded clade K. We aimed at achieving a dense geographic and taxonomic sampling of the expanded clade K. For this, we included 56 species previously recovered by Barfuss et al. (2016) and Granados Mendoza et al. (2017) within the expanded clade K, plus 37 additional *Tillandsia* subg. *Tillandsia* species mainly distributed in Central America and Mexico, which we hypothesized might belong in the expanded clade K given the geographic distribution pattern reported by Granados Mendoza et al. (2017). Sampling within *T.* subg. *Tillandsia* was complemented with three and five species previously recovered by Barfuss et al. (2016) within the *T. secunda* and *T. paniculata* clades, respectively, plus two additional species distributed in the Caribbean and South America and included here for the first time in a phylogenetic context, resulting in a total of 103 sampled species for *T.* subg. *Tillandsia*. Other subgenera and species complexes within *Tillandsia*, recognized by Barfuss et al. (2016), were sampled as follows: *T.* subg*. Aerobia* (8 spp.)*, T.* subg*. Anoplophytum* s. str. (5 spp.), *T.* subg*. Diaphoranthema* (5 spp.), *T.* subg*. Phytarrhiza* s. str. (3 spp.), *T.* subg*. Pseudovriesea* (2 spp.), *T.* subg*. Viridantha* (4 spp.), *T. australis* complex (1 sp.), *T. biflora* complex (21 spp.), *T. disticha* complex (1 sp.), *T. gardneri* complex (3 spp.), *T. purpurea* complex (1 sp.), *T. rauhii* complex (2 spp.), and *T. sphaerocephala* complex (5 spp.), as well as the unclassified species *T. albertiana* and *T. esseriana*. Tribe Tillandsieae was further represented by species of the genera *Barfussia* (2 spp.), *Guzmania* (2 spp.), *Lemeltonia* (1 sp.), *Pseudalcantarea* (2 spp.), *Racinaea* (3 spp.), and *Wallisia* (2 spp.). Within tribe Vrieseeae, subtribe Cipuropsidinae was represented by the genera *Goudaea* (2 spp.), *Lutheria* (2 spp.), *Mezobromelia* (1 sp.), *Werauhia* (3 spp.), and *Zizkaea* (1 sp.), whereas for subtribe Vrieseinae the genera *Alcantarea* (2 spp.), *Stigmatodon* (2 spp.), and *Vriesea* (4 spp.) were sampled. We included three species of the genus *Catopsis* as representatives of tribe Catopsideae. *Brocchinia micrantha* (subfamily Brocchinioideae) and *Typha latifolia* (Typhaceae) were sampled to represent the crown and stem nodes of Bromeliaceae, respectively. *Typha latifolia* was used to root the phylogenetic tree following the Poales familial phylogenetic relationships from the Kew Tree of Life Explorer (https://treeoflife.kew.org/). Lineages of Tillandsioideae not available to us include tribe Glomeropitcairnieae, and genera *Gregbrownia* (tribe Tillandsieae), *Josemania*, *Cipuropsis*, *Jagrantia*, and *Waltillia* (tribe Vrieseeae; Barfuss et al., 2016; Leme et al., 2017). Classification within Tillandsioideae follows Barfuss et al. (2016) and Gouda et al. (continuously updated), whereas the subfamilial classification of Bromeliaceae follows Givnish et al. (2007).

### DNA isolation, library preparation, enrichment, and sequencing

New plastomes were assembled from raw data derived from a Hyb-Seq approach that applies a modified version of the universal probe kit for angiosperms of Buddenhagen et al. (2016), which includes additional Bromeliaceae target sequences. A previous publication (Vera-Paz et al., 2022) demonstrated the feasibility of assembling complete plastomes from data derived from this Hyb-Seq project, and herein-employed molecular methods were according to these authors. DNA isolation was performed at the “Laboratorio de Biología Molecular, Laboratorio Nacional de Biodiversidad (LANABIO)” of the Institute of Biology of the National Autonomous University of Mexico (IB-UNAM). The DNeasy Plant Pro Kit (Qiagen) was used to isolate genomic DNA from silica-gel-dried leaf tissue. A minimum DNA concentration of 1.0 µg/µl and a DNA purity 260/280 ratio ≥1 was targeted. DNA concentration and purity were quantified with a Qubit™ fluorometer v. 3.0 (Invitrogen™ Thermo Fisher Scientific) using the Qubit™ dsDNA BR Assay Kit (Invitrogen™ Thermo Fisher Scientific) and a NanoDrop 2000 spectrophotometer (Thermo Scientific™), respectively. Genomic DNA molecular weight was assessed in 1% agarose test gels, ran at 85 V and 500 mA for 50 min. Library preparation, enrichment, and sequencing were carried out by Daicel Arbor Biosciences (https://arborbiosci.com/). There, DNA concentration was additionally assessed by spectrofluorimetric assays with a PicoGreen assay kit (Thermo Fisher Scientific). A Qsonica Q800 ultrasonicator was used to fragment genomic DNA to a target insert length of 300–800 nt. A proprietary modification by Daicel Arbor Biosciences of the KAPA HyperPrep kit (Roche) protocol was applied for library preparation, using custom unique dual-index combinations for the samples. Spectrofluorimetric assays and quantitative PCR assays using a KAPA Library Quantification Kit (Roche) were carried out to quantify the indexed libraries. Capture was carried out in pools of 12 libraries per reaction following the myBaits v. 5 protocol (https://arborbiosci.com/mybaits-v5-chemistry/). After capture, enrichment reactions were amplified for 12 cycles and quantified with spectrofluorimetric and quantitative PCR assays. Based on the number of libraries in each capture, the captures were pooled in approximately equimolar ratios and combined with equimolar pools of non-captured libraries at a 70:30 ratio. Sequencing was performed on the Illumina NovaSeq 6000 platform on S4 PE150 lanes targeting 2 Gbp of sequencing effort per library.

### Plastome assembly and annotation

In general, we followed the plastome assembly and annotation methods described in Vera-Paz et al. (2022). In short, raw read quality was visualized in FastQC v. 0.11.9 (https://www.bioinformatics.babraham.ac.uk/projects/fastqc/). Then, the reads’ leading and trailing low-quality or N bases as well as adapters were trimmed with Trimmomatic v. 39 (Bolger et al., 2014). Success of the trimming process was verified in FastQC. Trimmed reads were used to perform *de novo* assemblies in GetOrganelle v. 1.5.0 (Jin et al., 2020), applying default settings for 150 bp long paired data. In cases where GetOrganelle assembled partial plastome sequences as separated scaffolds, the SPAdes v. 3.15.5 (Prjibelski et al., 2020) assembler was used in an effort to try and expand the scaffolds further. Using *Tillandsia utriculata* (ON398158; Vera-Paz et al., 2022) as reference, annotations were automatically transferred to the newly assembled plastomes in Geneious Prime^®^ 2021.2.2 (https://www.geneious.com/) and manually curated. Inverted repeat regions were identified with the repeat finder plugin of Geneious Prime^®^. Newly sequenced and assembled full plastomes can be found in GenBank under the accession numbers OQ935587–OQ935748.

### Phylogenetic reconstruction and divergence time estimation

Full plastomes were aligned using the MAFFT v. 7.490 (Katoh and Standley, 2013) plugin of Geneious Prime^©^. The resulting alignment was manually curated and inspected to locate inversions. Gblocks v. 0.91b (Castresana, 2000) was then used to identify ambiguously aligned regions using default settings, except for the allowed gap position parameter which was set to the option “all.” Then, the -E command of RAxML v. 8.2.10 (Stamatakis, 2014) was applied to exclude: 1) small (<200 pb long) inversions, 2) ambiguously aligned regions detected by Gblocks, and 3) one of the inverted repeat regions. Long inversions (>200 pb long) detected in genus *Vriesea*, three species from the *Tillandsia biflora* complex clade N (i.e., *T. deppeana*, *T. heterophylla*, and *T. imperialis*), and *Werauhia gladioliflora*, were replaced by “?” in the species where they were present and considered as missing data. This modified alignment was partitioned by protein-coding genes, tRNA genes, rRNA genes, introns, and intergenic spacers (IGS) and analyzed in PartitionFinder v. 2.1.1 (Lanfear et al., 2016) to select the best-fit partitioning scheme and corresponding models of molecular evolution using the Bayesian Information Criterion (BIC).

Estimation of phylogenetic relationships and divergence times was carried out simultaneously in BEAST2 v. 2.6.1 (Bouckaert et al., 2014). In BEAUti v. 2.5, we set the analysis priors for two data partitions retrieved as the best-fit partitioning scheme by PartitionFinder, applying the GTR and GTR+G models of molecular evolution, respectively, and empirical base frequencies for both site models. An uncorrelated relaxed clock model was applied, with rates sampled from a log-normal prior (Drummond et al., 2006) and a birth-death model tree prior. Based on the phylogenetic relationships recovered by Vera-Paz et al. (2022) and to reduce the computational cost, the topology was constrained for the major clades Bromeliaceae, Tillandsioideae, Catopsideae, Vrieseeae, and Tillandsieae, leaving unconstrained relationships among and within these clades. Given the lack of fossils that can be unambiguously assigned to Bromeliaceae and dated with confidence (e.g., Baresch et al., 2011), three secondary calibrations were applied using uniform prior distributions obtained from the relaxed (conservative fossil set) calibration performed by Ramírez-Barahona et al. (2020), as follows: 1) the root of the tree, i.e., stem node of Bromeliaceae (lower: 90.8908 Ma, upper: 123.4522 Ma), 2) the crown node of Bromeliaceae (lower: 15.3615 Ma, upper: 37.7002 Ma), and 3) the crown node of Tillandsioideae (lower: 7.3867, upper: 19.3439). Seven independent Markov Monte Carlo (MCMC) chains were run for 271–300 million generations each, sampling every 5,000 steps, until convergence was achieved, which was verified in Tracer v1.7.1 (Suchard et al., 2018). MCMC chain results were summarized with LogCombiner v2.6.1, setting the burn-in to 10% and sampling every 50,000 trees. The maximum clade credibility (MCC) tree was generated in TreeAnnotator v. 2.6.0 and visualized in FigTree v1.4.4. A second phylogenetic analysis without topological constraints was carried out under maximum likelihood in IQ-TREE (Nguyen et al., 2015) with an automatic selection of the best-fit substitution model for the individual partitions and assessing node support with 1,000 ultrafast bootstrap replicates.

### Estimation of biogeographic history

#### Geographic data compilation and delimitation of distribution areas

The geographic distribution of the species included in our phylogenetic context was obtained from various resources including online public repositories (e.g., https://www.gbif.org/ and https://www.jacq.org/), taxonomic literature, own field records, and the study of Zizka et al. (2020; **Supplementary Material 2**). In the case of public databases, we restricted our search to records that could be associated with an image (e.g., photographs of herbarium vouchers), so that taxonomic identification could be verified if needed. Depending on the data source, geographic distribution data was retrieved as geographic coordinates, distribution polygons (as in Plants of the World Online, https://powo.science.kew.org/), and description of localities.

Geographic coordinates were filtered in R v. 4.2.1 (R Core Team, 2022) and R studio v. 2022.12.0.353 (Posit team, 2022) with the packages *maps* v. 3.4.1 (Becker et al., 2022), *dplyr* v. 1.1.0 (Wickham et al., 2023), *raster* v. 3.6-14 (Hijmans, 2023), *rgdal* v. 1.6-4 (Bivand et al., 2023), and *sp* (Pebesma and Bivand, 2005; Bivand et al., 2013) by plotting them on the map of America then filtering out coordinates falling in the sea, duplicated values, and coordinates that did not match their associated locality description. After this data filtering, the distributional range of every species was validated with the ArcMap tool of ArcGIS v. 10.5 using the Bromeliaceae species distribution polygons modelled by Zizka et al. (2020) as reference, which were obtained with the R package *speciesgeocodeR* (https://github.com/azizka/speciesgeocodeR). Validation of species lacking a distribution polygon in Zizka et al. (2020) was performed manually based on the geographic distribution reported in taxonomic literature, as well as the online databases Encyclopaedia of Bromeliads v. 4 (http://bromeliad.nl/encyclopedia/) and Plants of the World Online (https://powo.science.kew.org/).

We estimated ancestral areas based on the biogeographic regionalization of the Neotropical region proposed by Morrone et al. (2019; 2022) and the terrestrial ecoregions of North America (https://www.epa.gov/eco-research/ecoregions-north-america). Because analysis of a high number of areas can hinder the interpretation of biogeographic reconstructions, we performed two separate analyses to infer both the origin and dispersal history of our focal group, the expanded clade K. The first analysis, designed for reconstructing the origin of the focal group, was performed on the dated phylogeny including the complete taxon sampling and the following general discrete areas: Chacoan subregion (A), Boreal and South Brazilian dominions (B), South American transition zone (C), Pacific dominion (D), Antillean subregion + SE USA (E), and Mexican transition zone + Mesoamerican dominion + Nearctic region (F). The second analysis aimed at reconstructing the biogeographic history of the expanded clade K. For this, a subtree corresponding to *Tillandsia* subg. *Tillandsia* was extracted from the original dated phylogeny and analyzed with the following specific discrete areas: Pacific and Boreal and South Brazilian dominions + South American transition zone (Y), Antillean subregion + SE USA (E), Pacific Lowlands and Balsas provinces + El Cabo de Baja California district (G), Mexican transition zone (H), Mosquito, Veracruzan, and Yucatán Peninsula provinces (I), and Nearctic region (J). The ArcMap tool of ArcGIS v. 10.5 was used to delimit the above proposed areas and to convert them into shapefiles. These shapefiles were plotted along with the compiled species geographic distribution information (i.e., polygons, geographic coordinates, and georeferenced localities) to extract the species presence in each area with the tool “select layer by location” of ArcMap. The codification of the distribution areas for all the sampled species can be found in the **Supplementary Material 2**.

#### Ancestral area reconstructions

Both ancestral area reconstruction analyses were performed in R v. 4.1.1 and R studio v. 1.4.17.17 with the package BioGeoBEARS 0.2.1 (Matzke, 2013). First, widely distributed species, namely, *Catopsis sessiliflora*, *Tillandsia balbisiana*, *T. fasciculata*, *T. juncea*, *T. polystachia*, *T. pruinosa*, *T. recurvata*, *T. usneoides*, and *T. utriculata*, were excluded in RASP v4.2 (Yu et al., 2015) with the tool “remove selected groups” to avoid reconstructing an excessive number of ancestral areas. Then, we tested the relative fit of the data to the biogeographic models Dispersal-Extinction-Cladogenesis (DEC), Dispersal-Vicariance-Like (DIVALIKE), BayArea-like (BAYAREALIKE), and the same models plus the founder-event speciation (*j*) parameter. The *j* parameter was considered in model comparison because Tillandsioideae species have seeds with hair-like appendages that form a flight apparatus (wind-dispersed seeds; Barfuss et al., 2016), suggesting that founder-event speciation is a factor potentially influencing the biogeographic history of this group. For all models, we set the maximum number of areas that any species could occupy to five and allowed *j*, as well as the rate of dispersal/range addition (*d*) and rate of extinction/range contraction (*e*) parameters, to vary freely.

Selection of the best-fitting biogeographic model was performed in a likelihood framework, using the Akaike’s information criterion weight (AIC-wt) and contrasting the original models versus these models + *j* as alternative hypotheses with the AICstats_2models function of BioGeoBEARS. Ancestral area probabilities reconstructed under the best-fitting biogeographical model for each node of the analyzed phylogenies were printed to a CSV file, along with a tree with reference node number labels (**Supplementary material 2** and **3**). The most probable areas were plotted at the nodes of the dated phylogenies in R. Phylogenetic inference, dating, and ancestral area reconstruction were performed on the servers BEAGLE of IB-UNAM and CIPRES Science Gateway (Miller et al., 2010).

## Results

### Phylogenetic relationships

**Figure 2:**
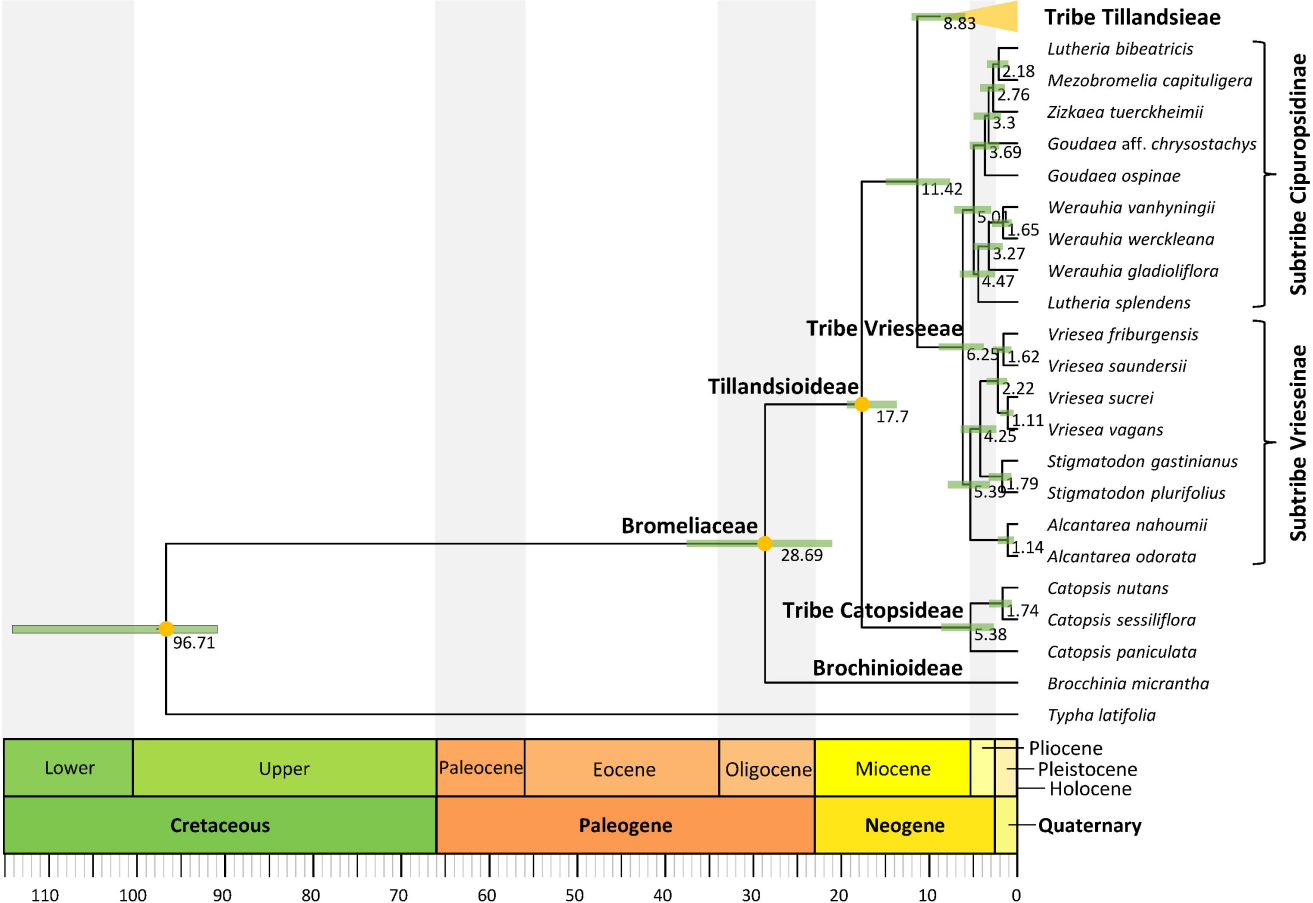
Tillandsioideae tribe and subtribe phylogenetic relationships. Maximum clade credibility tree derived from the analysis of full plastomes. Green bars associated with age values are the 95% highest posterior density (HPD) intervals of highly supported (PP ≥0.85) nodes. Yellow circles denote calibrated nodes. Clade names follow Barfuss et al. (2016) and Givnish et al. (2007).

**Figure 3:**
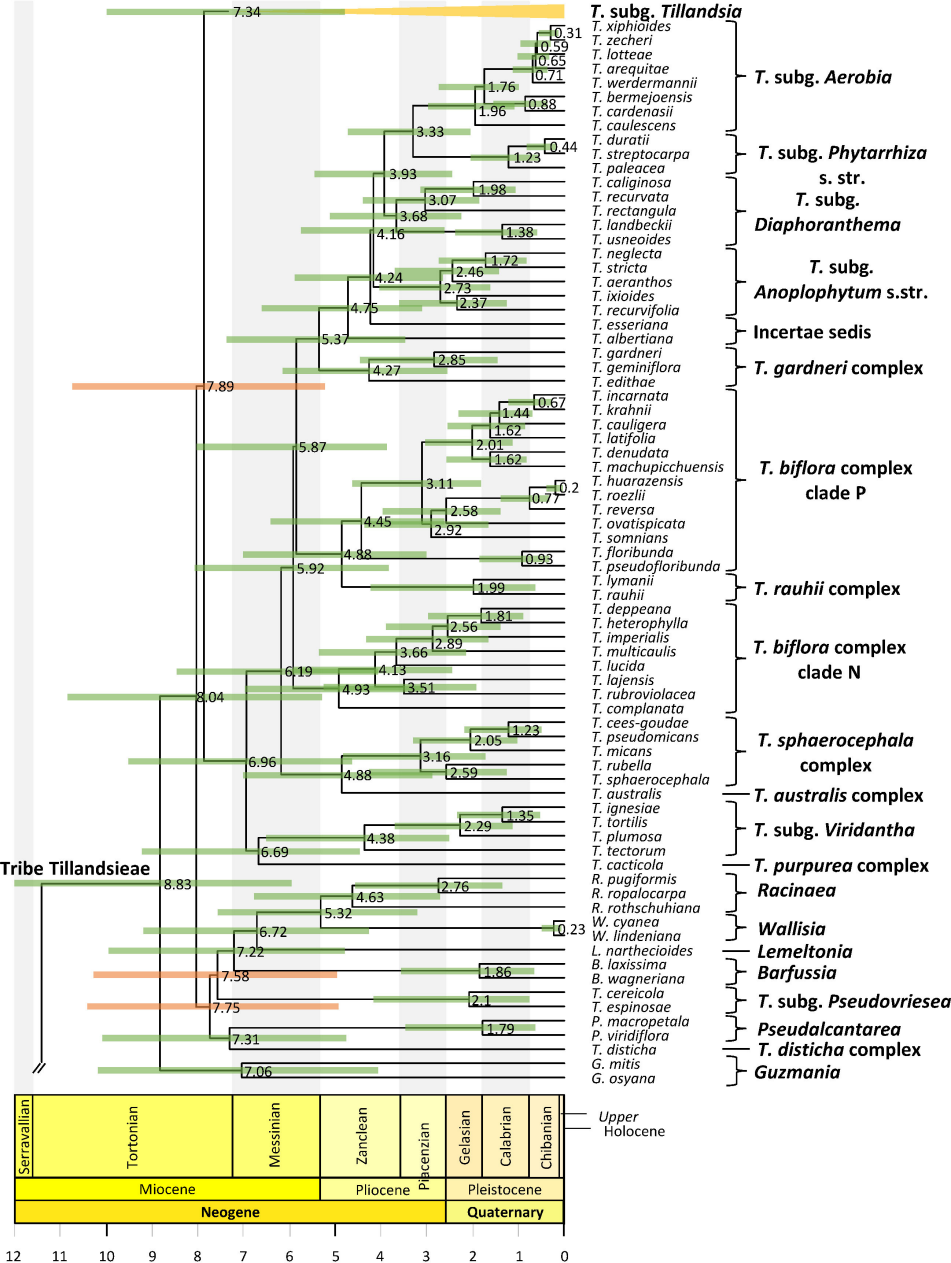
Tribe Tillandsieae phylogenetic relationships. Maximum clade credibility tree derived from the analysis of full plastomes. Bars associated with age values are the 95% highest posterior density (HPD) intervals. Green and orange HPD bars denote highly supported (PP ≥0.85) and non-highly supported (PP ≤0.85) nodes, respectively. Clade names follow Barfuss et al. (2016).

**Figure 4:**
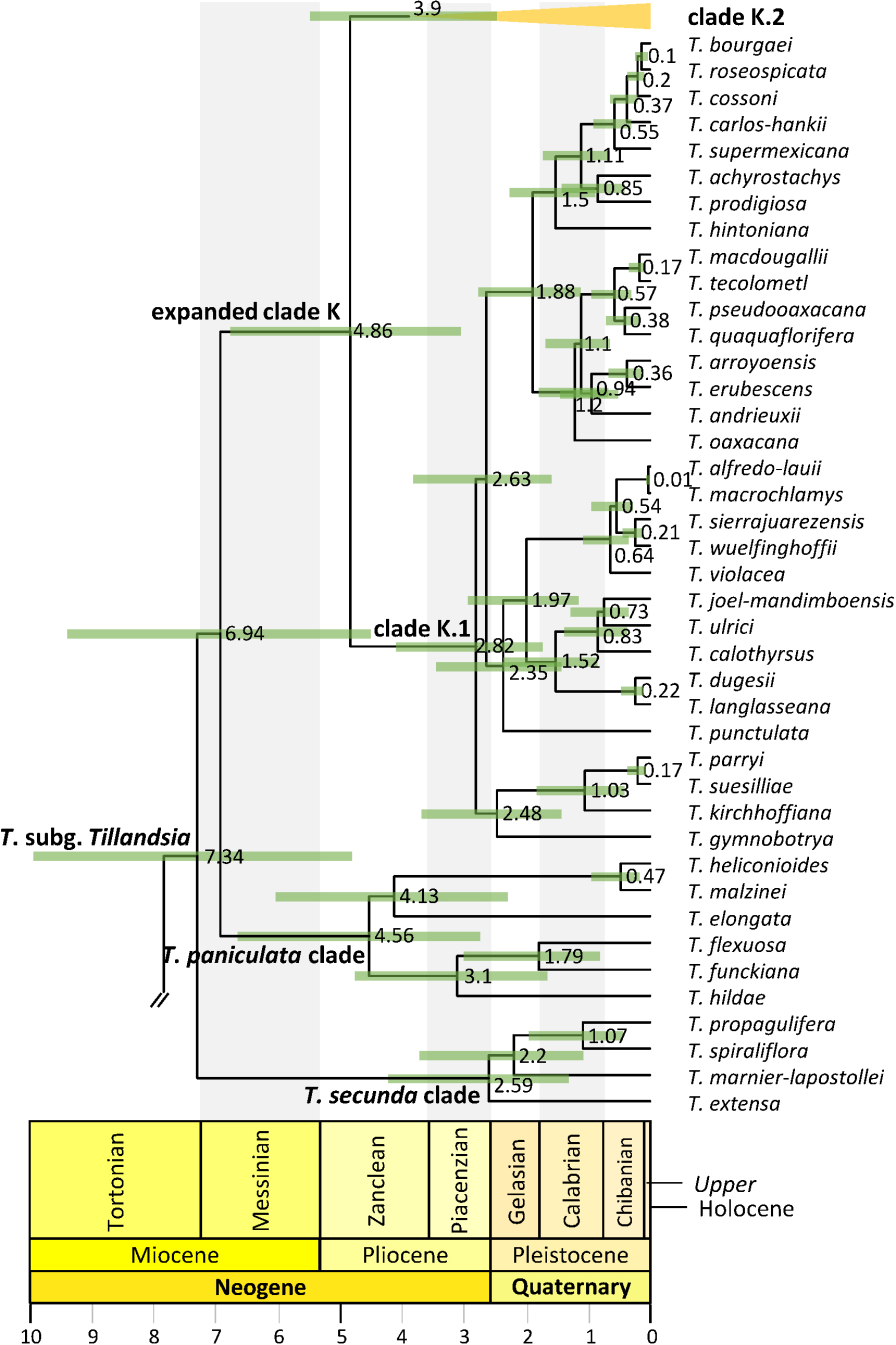
*Tillandsia* subg. *Tillandsia* phylogenetic relationships. Maximum clade credibility tree derived from the analysis of full plastomes. Green bars associated with age values are the 95% highest posterior density (HPD) intervals of highly supported (PP ≥0.85) nodes. Clade names follow Barfuss et al. (2016) and Granados Mendoza et al. (2017).

**Figure 5:**
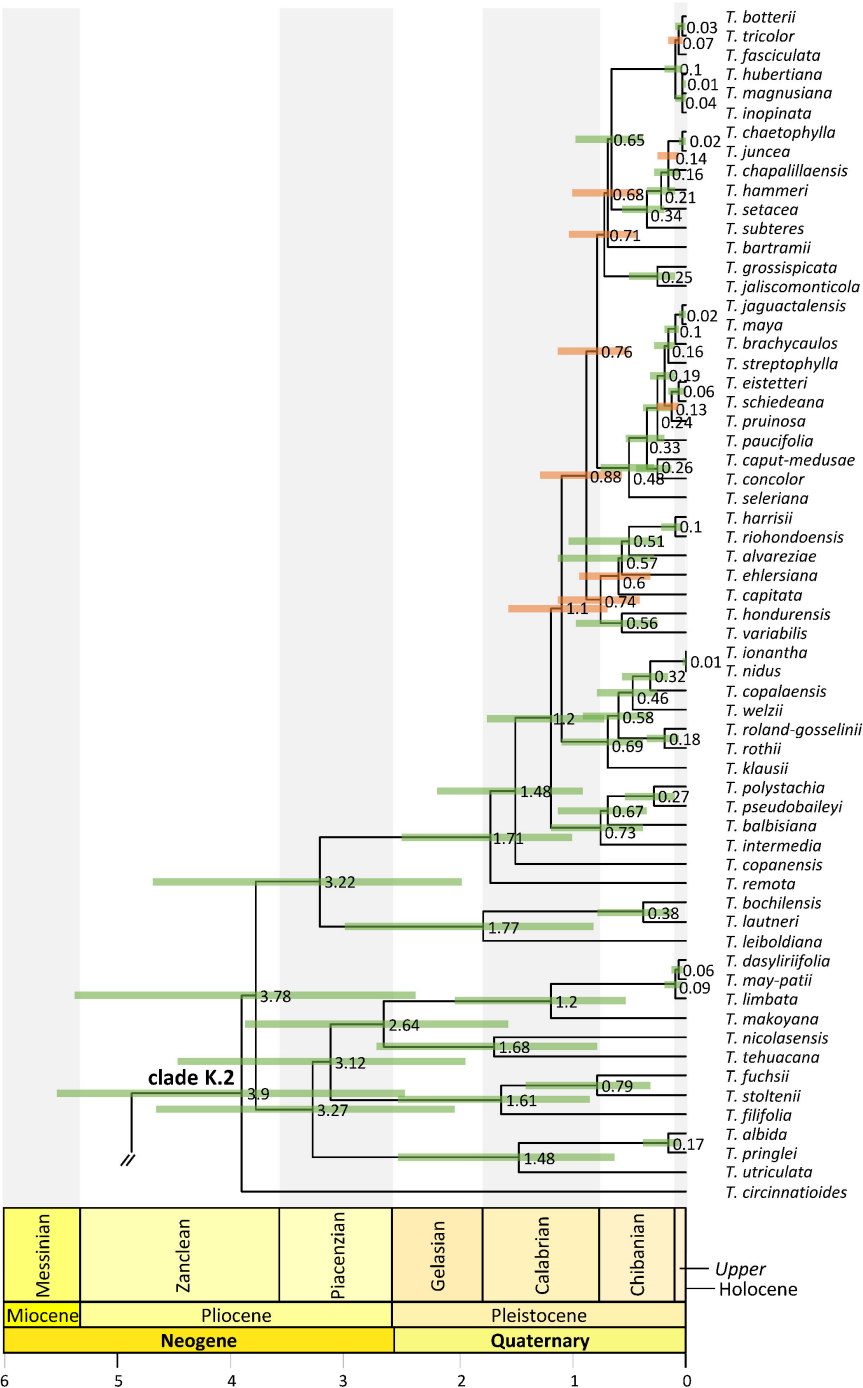
Clade K.2 phylogenetic relationships. Maximum clade credibility tree derived from the analysis of full plastomes. Bars associated with age values are the 95% highest posterior density (HPD) intervals. Green and orange HPD bars denote highly supported (PP ≥0.85) and non-highly supported (PP ≤ 0.85) nodes, respectively. Clade names follow Granados Mendoza et al. (2017).

The alignment of the complete plastomes resulted in a 180,766 bp matrix. A total of 48,040 bp corresponding to one inverted repeat region, small inversions, and ambiguously aligned regions were excluded resulting in a 132,726 bp long matrix that was used for phylogenetic inference. Nearly all phylogenetic relationships of the BEAST2 analysis received strong statistical support (PP ≥0.85), excepting as noted below (**Figures 2**–**5**). Genera, subgenera, and species complexes from which we included more than one accession were recovered as monophyletic, except for *Goudaea*, *Lutheria*, *Tillandsia*, and the *T. biflora* complex. Tribe Catopsideae was recovered as the sister lineage of tribes Vrieseeae plus Tillandsieae. In subtribe Vrieseinae, *Alcantarea* is sister of a clade including *Stigmatodon* and *Vriesea*, whereas subtribe Cipuropsidinae is further divided in two lineages, one including *Lutheria splendens* as sister of *Werauhia* and the other including *Goudaea ospinae* as successive sister of *Goudaea* aff. *chrysostachys*, *Zizkaea*, *Mezobromelia*, and *Lutheria bibeatricis* (**Figure 2**). Within tribe Tillandsieae, *Guzmania* is the sister lineage of a clade containing the remaining sampled species, which is divided into two main lineages. The first of these lineages (PP = 0.60) consists of a clade including the *Tillandsia disticha* complex and *Pseudalcantarea* as successive sisters (PP = 0.74) of *T*. subg. *Pseudovriesea*, *Barfussia*, *Lemeltonia*, and *Wallisia* plus *Racinaea*. The second lineage (PP = 0.34) is further divided into two clades, the first of them including a clade of *T. purpurea* complex and *T*. subg. *Viridantha* as the successive sister of 1) a clade including *T. australis* and *T. sphaerocephala* complexes, 2) the *T. biflora* complex clade N, 3) a clade including the *T. rauhii* complex and the *T. biflora* complex clade P, 4) the *T. gardneri* complex, 5) *T. albertiana*, 6) *T. esseriana*, 7) *T*. subg. *Anoplophytum* s. str., 8) *T*. subg. *Diaphoranthema*, and 9) a clade of *T*. subg. *Aerobia* and *T*. subg. *Phytarrhiza* s. str. (**Figure 3**). The second clade corresponds to *T.* subg. *Tillandsia*, where all but a few recent and shortly spaced apart nodes within the later mentioned clade K.2 were strongly supported (**Figures 4**, **5**). Within *T.* subg. *Tillandsia*, three main clades were recovered as successive sister lineages (**Figure 4**). In the first of them, *T. extensa* is the sister species of a clade composed of *T. marnier-lapostollei*, *T. spiraliflora*, and *T. propagulifera* (corresponding to *T. secunda* clade). The second main clade, *T. paniculata* clade, includes two lineages, the first one including *T. hildae* as sister of *T. funckiana*–*T. flexuosa*, whereas in the second *T. elongata* is sister to *T. malzinei*–*T. heliconioides*. The third main clade is highly diverse, herein including 93 spp., equivalent to the expanded clade K, which consists of two main clades herein identified as K.1 (31 spp.) and K.2 (62 spp.) following the clade nomenclature proposed by Granados Mendoza et al. (2017). Given the substantial number of species integrating these two clades, internal phylogenetic relationships will not be described in detail, but they are shown in full in **Figures 4**, **5**. When considering only strongly supported relationships (BS ≥85 and PP ≥0.85), the ML analysis resulted in a tree topology almost entirely congruent with the BEAST2 analysis, except for the alternative sister position of *T. somnians* and *T. ovatispicata* relative to the clade of *T. huarazensis*, *T. roezlii*, and *T. reversa*, as well as the position of *Goudaea* aff. *chrysostachys* as sister of *Mezobromelia capituligera* instead of sister to the clade of *Mezobromelia capituligera*, *Lutheria bibeatricis*, and *Zizkaea tuerckheimii*.

### Lineage divergence time

Divergence between Bromeliaceae and Typhaceae was estimated to have occurred 96.71 Mya (95% HPD = 90.89–114.14 Mya), whereas estimated crown ages of Bromeliaceae and Tillandsioideae were 28.69 Mya (95% HPD = 21.06–37.58 Mya) and 17.7 Mya (95% HPD = 13.72–19.34 Mya), respectively. Tribe Catopsideae, with a crown age of 5.38 Mya (95% HPD = 2.73–8.69 Mya), diverged from tribes Vrieseeae and Tillandsieae 17.7 Mya (95% HPD = 13.72–19.34 Mya), whereas divergence of the latter two tribes was 11.42 Mya (95% HPD = 7.66–15.01 Mya). The crown age of tribe Vrieseeae was 6.25 Mya (95% HPD = 3.81–8.98 Mya), whereas that of its subtribes Vrieseinae and Cipuropsidinae were 5.39 Mya (95% HPD = 3.16–7.92 Mya) and 5.01 Mya (95% HPD = 2.97–7.25 Mya), respectively (**Figure 2**). The crown age of tribe Tillandsieae was 8.83 Mya (95% HPD = 5.94–12 Mya), and within it, *Tillandsia* subg. *Tillandsia* had stem and crown ages of 7.89 (95% HPD = 5.22–10.73 Mya) and 7.34 Mya (95% HPD = 4.8–9.98 Mya), respectively (**Figure 3**). The stem and crown ages of the expanded clade K focal group were 6.94 (95% HPD = 4.49–9.42 Mya) and 4.86 Mya (95% HPD = 3.03–6.79 Mya), respectively, whereas the crown ages of its two subclades K.1 and K.2 were 2.82 (95% HPD = 1.72–4.1 Mya) and 3.9 Mya (95% HPD = 2.45–5.5 Mya), respectively (**Figures 4**, **5**). The estimated stem and crown ages of other sampled genera and species complexes are shown in **Supplementary Material 4**, and point-age estimations for all recovered nodes are shown in **Figures 2**–**5**.

### Biogeographic history

**Figure 6:**
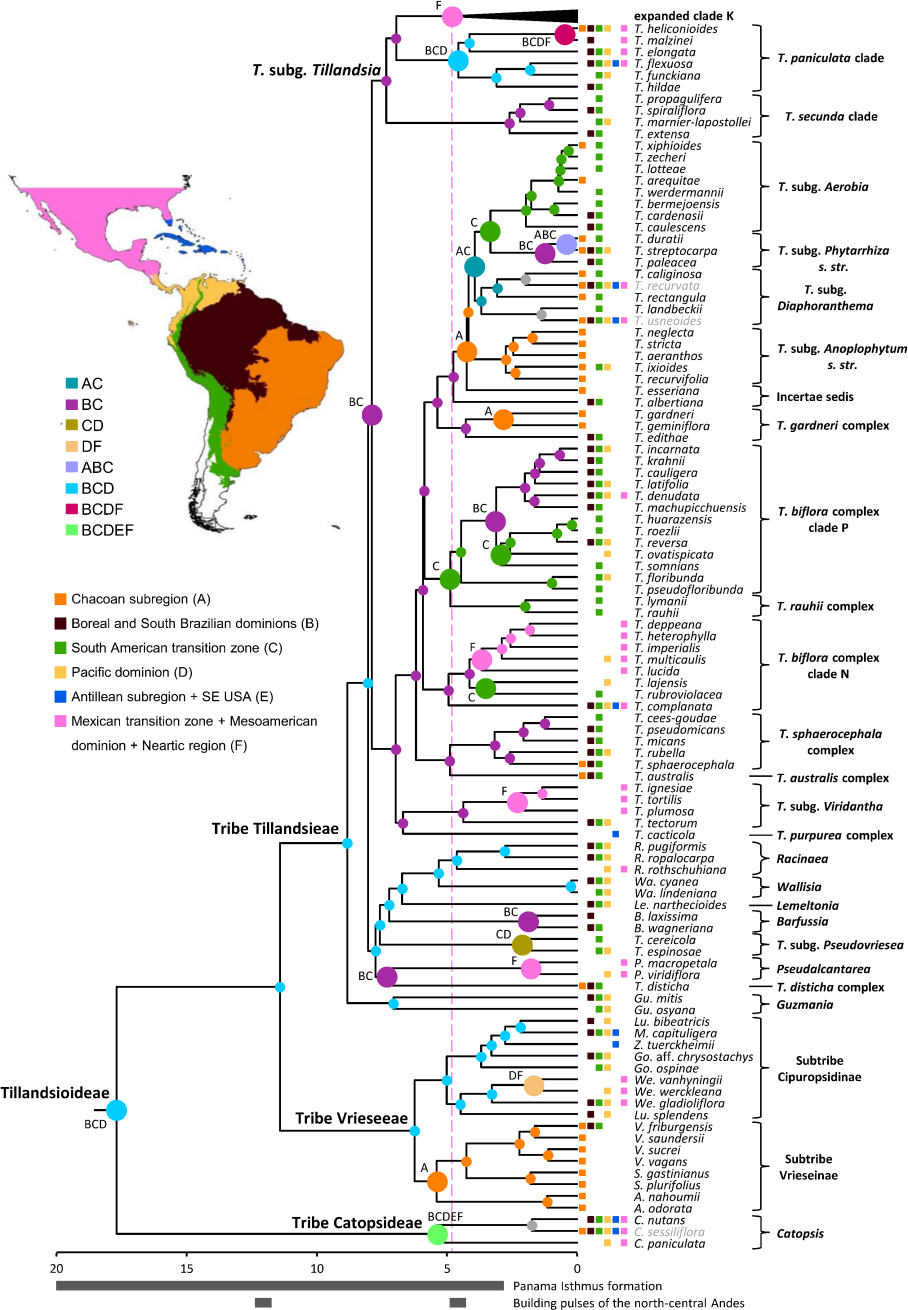
Geographic origin of the focal group expanded clade K. Colored circles denote the most probable individual or combined ancestral area under the BAYAREALIKE+J model for highly supported (PP ≥ 0.85) internal nodes. Larger circles denote area shifts. Current geographic distribution of the species is denoted by colored squares. Excluded cosmopolitan species and their associated nodes are shown in gray. Proposed periods of main tectonic events discussed in the text are denoted by gray bars below time scale. Clade names follow Barfuss et al. (2016); Givnish et al. (2007), and Granados Mendoza et al. (2017). Source of map: http://tapiquen-sig.jimdo.com (Carlos Efraín Porto Tapiquén. Orogénesis Soluciones Geográficas. Porlamar, Venezuela 2015. Based on layers from the Enviromental Systems Research Institute - ESRI).

Log likelihoods and estimated *j*, *d*, and *e* parameter values for each tested biogeographic model for both analyses are shown in **Table 1**. The model that best fitted the data in both cases is BAYAREALIKE+*j*, explaining ca. 99% and 72% of the total predictive power of all assessed models in the analyses with the complete taxon sampling and *Tillandsia* subg. *Tillandsia*, respectively. Among all analyzed areas, the combined area of the Boreal and South Brazilian dominions (B), South American transition zone (C), and Pacific dominion (D) was recovered as the most probable ancestral area for the nodes corresponding to subfamily Tillandsioideae (8%), core Tillandsioideae (45%), tribe Tillandsieae (59%), and a node including all tribe Tillandsieae representatives except *Guzmania* (59.20%). Then, the three nodes preceding the expanded clade K, including that of *T.* subg. *Tillandsia* (53%), were reconstructed as having experienced a range contraction to the Boreal and South Brazilian dominions (B) and South American transition zone (C), followed by a non-contiguous area shift to the Mexican transition zone + Mesoamerican dominion + Nearctic region individual area (F; 98.60%). Within *T.* subg. *Tillandsia*, the node subtending the *T. secunda* clade is similarly reconstructed in the combined area of the Boreal and South Brazilian dominions (B) and South American transition zone (C; 78.60%), whereas the *T. paniculata* clade expanded its ancestral area back to the Pacific dominion (D; 48.50%; **Figure 6**, see **Supplementary Material 3C** for the second most probable ancestral area).

**Table 1:** Tested biogeographic models are Dispersal-Extinction-Cladogenesis (DEC), Dispersal-Vicariance-Like (DIVALIKE), and BayArea-like (BAYAREALIKE) with and without the founder–event speciation (*j*) parameter.

Models	LnL	*d*	*e*	*j*	AIC	AIC-wt
Full sampling analysis						
DEC DEC+*j*	-461.300 -461.300 *p* = 1	0.521 0.790	0.212 0.958	0 1.0E-05	926.600 928.600	0.730 0.270
DIVALIKE DIVALIKE+*j*	-486.800 -486.800 *p* = 1	1.036 1.591	1.526 2.015	0 1.0E-05	977.600 979.600	0.730 0.270
BAYAREALIKE **BAYAREALIKE*+j***	-465.400 **-459.400** *p* = 5.0E-04	0.081 0.085	0.220 0.239	0 0.005	934.800 924.800	0.007 0.993
*Tillandsia* subg. *Tillandsia* analysis						
DEC DEC+*j*	-464.300 -446.800 *p* = 3.3E-9	0.048 0.048	0.015 0.015	0 1.00E-05	932.600 899.600	6.8E-08 0.999
DIVALIKE DIVALIKE+*j*	-467.400 -463.600 *p* = 0.006	0.054 0.054	0.021 0.021	0 1.00E-05	938.700 933.100	0.058 0.942
BAYAREALIKE **BAYAREALIKE+*j***	-357.000 **-355.100** *p* = 0.049	0.035 0.027	0.087 0.057	0 1.00E-05	718.00 716.200	0.280 0.720

The rate of dispersal/range addition (d) and rate of extinction/range contraction (e) parameters were allowed to vary freely. Resulting log-likelihood (LnL) are indicated for each model and that of the best-fitting model is highlighted in bold for each analysis as selected by the Akaike’s information criterion weight (AICwt).

**Figure 7:**
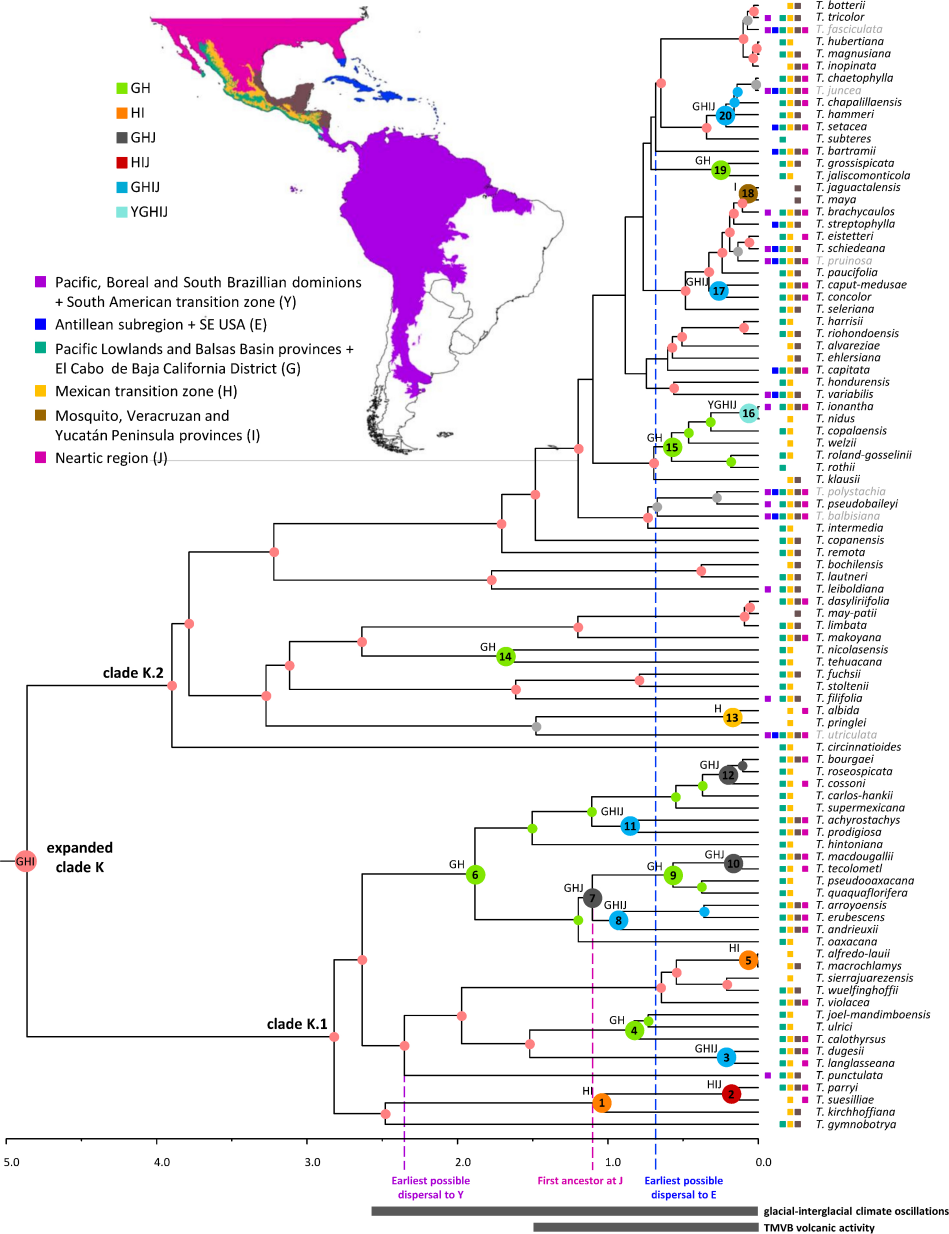
Dispersal history of the focal group expanded clade K. Colored circles denote the most probable individual or combined ancestral area under the BAYAREALIKE+J model for highly supported (PP ≥ 0.85) internal nodes. Larger circles at internal nodes denote area shifts. Node numbers correspond to those discussed in the text. Current geographic distribution of the species is denoted by colored squares. Excluded cosmopolitan species and their associated nodes are shown in gray. Proposed periods of main tectonic events discussed in the text are denoted by gray bars below time scale. Clade names follow Granados Mendoza et al. (2017). Source of Map: http://tapiquen-sig.jimdo.com (Carlos Efraín Porto Tapiquén. Orogénesis Soluciones Geográficas. Porlamar, Venezuela 2015. Based on layers from the Enviromental Systems Research Institute - ESRI).

Key nodes relevant for the biogeographic discussion of the expanded clade K are indicated in **Figure 7** with the numbers 1–20 (see **Supplementary Material 3D** for the second most probable ancestral area). The most recent common ancestor of this focal group is estimated to have occupied the combined area of the Pacific Lowlands and Balsas Basin provinces + El Cabo de Baja California district (G), Mexican transition zone (H), and the Mosquito, Veracruzan, and Yucatán Peninsula provinces (I; 31%). From this ancestral area, four lineages within clade K.1 were reconstructed to have reduced their range to either the Mexican transition zone (H) and the Mosquito, Veracruzan, and Yucatán Peninsula provinces (I; node 1 and 5; 36% and 89%) or to the Pacific Lowlands and Balsas Basin provinces + El Cabo de Baja California district (G) and the Mexican transition zone (H; nodes 4 and 6; 50% and 38%), whereas one lineage experienced a range expansion to the Nearctic region (J; node 3; 53%). Further colonization of the Nearctic region (J) occurred *via* additional area range expansions from the Mexican transition zone (H) and the Mosquito, Veracruzan, and Yucatán Peninsula provinces (I; node 2, 47%), as well as the Pacific Lowlands and Balsas Basin provinces + El Cabo de Baja California district (G) and the Mexican transition zone (H; nodes 7, 12, 28% and 62%). Additional dispersals to the Mosquito, Veracruzan, and Yucatán Peninsula provinces (I) involved range expansions from the combined area of the Pacific Lowlands and Balsas Basin provinces + El Cabo de Baja California district (G), the Mexican transition zone (H), and the Nearctic region (J; node 8, 54%) or from the Pacific Lowlands and Balsas Basin provinces + El Cabo de Baja California district (G) and the Mexican transition zone (H), however, with a simultaneous area expansion to the Nearctic region (J, node 11, 54%). Successive area contraction and expansion were modeled at nodes 9 (47.9%) and 10 (80%) in combined areas involving the Pacific Lowlands and Balsas Basin provinces + El Cabo de Baja California district (G), the Mexican transition zone (H), and the Nearctic region (J). Within clade K.1, a single area range expansion back to the Pacific, Boreal and South Brazilian dominions + South American transition zone (Y) is reconstructed in a terminal branch for *Tillandsia punctulata*.

Ancestral area reconstruction within clade K.2 is only described for nodes that received strong statistical support (PP ≥0.85) in the phylogenetic analysis. Within this clade, several range contractions were reconstructed to either the Pacific Lowlands and Balsas Basin provinces + El Cabo de Baja California district (G) and the Mexican transition zone (H; nodes 14, 15, and 19; 74%, 72.50%, and 83.88%); the Mexican transition zone (H; node 13, 80.20%); or the Mosquito, Veracruzan, and Yucatán Peninsula provinces (I; node 18, 99%). Expansion to the Nearctic region (J) in clade K.2 occurred at nodes 17 (80%) and 20 (79%) from the ancestral area of the expanded clade K and from the Pacific Lowlands and Balsas Basin provinces + El Cabo de Baja California district (G) and the Mexican transition zone (H; node 16, 57%), however, with a simultaneous area range expansion to the Mosquito, Veracruzan, and Yucatán Peninsula provinces (I) and back to the Pacific, Boreal, and South Brazilian dominions + South American transition zone (Y). Eight additional area range expansions back to the Pacific, Boreal, and South Brazilian dominions + South American transition zone (Y) were estimated at terminal branches of clade K.2 corresponding to *Tillandsia filifolia*, *T. leiboldiana*, *T. pseudobaileyi*, *T. ionantha*, *T. variabilis*, *T. schiedeana*, *T. brachycaulos*, and *T. tricolor*. Colonization of the Antillean subregion + SE USA (E) occurred exclusively within clade K.2 in six independent events from either the combined area of the Pacific Lowlands and Balsas Basin provinces + El Cabo de Baja California district (G), Mexican transition zone (H), and the Mosquito, Veracruzan, and Yucatán Peninsula provinces (I, *T. schiedeana*, *T. variabilis*, and *T. streptophylla*), from the latter combined area plus the Nearctic region (J; *T. setacea*) or from an unknown area due to low statistical support of the phylogenetic relationships (*T. bartramii* and *T. capitata*).

## Discussion

### Evolutionary context of the expanded clade K

Our study contributes 162 newly sequenced and assembled complete plastomes to Bromeliaceae genomic resources, more densely sampled within the expanded clade K of *Tillandsia* subg. *Tillandsia* but also representing many other *Tillandsia* subgenera and species complexes, as well as other genera of Tillandsioideae. Additionally, the complete plastome of *Brocchinia micrantha* (subfamily Brocchinioideae) was generated here as part of our outgroup sampling. Our analyses of 200 complete plastomes derived in an overall strongly supported phylogenetic hypothesis, with only 13 out of the 198 nodes (excluding the root) with PP <0.85. At tribe and subtribe levels, our results agree with previously proposed plastid phylogenetic contexts (Barfuss et al., 2016; Machado et al., 2020; Loiseau et al., 2021; Vera-Paz et al., 2022). As in the plastid-based tree of Loiseau et al. (2021), we here recovered the genus *Goudaea* (subtribe Cipuropsidinae) as non-monophyletic (see also **Supplementary Material 5**), albeit herein with strong statistical support. However, the nuclear-based inference of Loiseau et al. (2021) supports the monophyly of this genus. Interestingly, those authors propose a potential hybrid origin for *Goudaea chrysostachys*, which could potentially explain the discordance between nuclear and plastid data sources across studies. The genus *Lutheria* is also non-monophyletic in our phylogenetic context with strong statistical support. Remarkably, the alternative phylogenetic positions shown by the herein analyzed *Lutheria* species (*L. splendens* and *L. bibeatricis*) follow the same alternative positions proposed by Loiseau et al. (2021) for this genus upon comparing their plastid and nuclear trees. Our study is the first testing the phylogenetic position of *L. bibeatricis*; therefore, its affiliation to the genus *Lutheria* and the reasons (e.g., hybridization) for its unexpected phylogenetic position should be further investigated. Other non-monophyletic groups recovered in our analysis with strong statistical support are the genus *Tillandsia* and the *T. biflora* complex. The non-monophyly of *Tillandsia* results from the sister relationship of *T. disticha* and *Pseudalcantarea*, since the obtained sister relationship of *T.* subg. *Pseudovriesea* to the clade including *Barfussia*, *Racinaea*, *Wallisia*, and *Lemeltonia* received low statistical support. A comparatively long branch subtends *T. disticha* in a maximum likelihood tree (**Supplementary Material 5**), a result shared with previous studies (e.g., Barfuss et al., 2016), suggesting that the phylogenetic position of this species could also be influenced by long branch attraction, leaving open the question if *Tillandsia*, as circumscribed by Barfuss et al. (2016), is or is not monophyletic. Additional statistical support is required to resolve recalcitrant nodes along the backbone of Core Tillandsieae (genus *Tillandsia* s.l., Smith and Downs, 1977) to validate or refute the segregation of various genera from *Tillandsia* s.l. (Grant and Zijlstra, 1998; Smith and Till, 1998; Barfuss et al., 2016). Since its recognition in Barfuss et al. (2016), the *T. biflora* complex (136 spp.) was defined as a polymorphic assemblage of species with contrasting geographic distributions, highlighting the presence of Caribbean and Mexican species as part of this complex. Although highly supported in the Bayesian analysis of Barfuss et al. (2016), the sister relationship of the *T. biflora* clades N and P received low statistical support in their maximum likelihood and parsimony analyses, which is in favor of the non-monophyly of this group. Also, *Tillandsia biflora* complex clades N and P show distinct biogeographical patterns, discussed below. The herein obtained phylogenetic relationships could help define which morphological attributes characterize these two independent lineages.

Three strongly supported lineages in successive sister relationship conform *Tillandsia* subg. *Tillandsia*, namely, the *T. secunda* clade, the *T. paniculata* clade, and the expanded clade K. These results are consistent with Barfuss et al. (2016), except that these authors recovered the *T. secunda* clade as the sister lineage of the expanded clade K instead of the *T. paniculata* clade as in our phylogeny. While we obtained strong statistical support for these relationships, Barfuss et al. (2016) obtained low statistical support in the ML and parsimony analyses, albeit a PP = 0.85 in the Bayesian analysis. The expanded clade K and its internal clades K.1 an K.2 have consistently been recovered as strongly supported monophyletic groups in the previous (Chew et al., 2010; Sidoti, 2015; Barfuss et al., 2016; Pinzón et al., 2016; Montes-Azcué, 2020; Vera-Paz et al., 2022; Yardeni et al., 2022) and present studies. Furthermore, species relationships and composition of clades K.1 and K.2 are congruent across previous studies (Sidoti, 2015; Barfuss et al., 2016; Pinzón et al., 2016; Montes-Azcué, 2020; Vera-Paz et al., 2022; Yardeni et al., 2022) and our study. While all internal relationships of the less diverse clade K.1 are strongly supported, several relationships within clade K.2, which contains the type species of the genus *Tillandsia*, *T. utriculata*, are subtended by short branches and lack strong statistical support. Resolution of these low supported phylogenetic relationships would require the use of more variable sequence data, such as low- or single copy nuclear loci. Although the *mat*K-*trn*K region analysis of Granados Mendoza et al. (2017) included a dense taxon sampling of the expanded clade K, internal resolution of clades K.1 and K.2 was extremely limited. Other studies focused on particular species complexes or groups, such as the *T. ionantha* complex (Ancona et al., 2022), pseudobulbous species (Chew et al., 2010), the *T. erubescens* complex (Granados Mendoza, 2008; Martínez-García et al., 2022), the *T. utriculata* complex (2016), and the *T. fasciculata* complex (Sidoti, 2015), used combinations of Sanger-sequenced DNA markers and/or morphology. When considering strongly supported phylogenetic relationships, our study is in overall agreement with plastid-based phylogenetic relationships recovered in those previous studies. However, there are some disagreements with the nuclear ribosomal phylogenetic context of Chew et al. (2010), who recovered a pair of species from clade K.2 (*T. pseudobaileyi*) and K.1 (*T. achyrostachys*) as sister to one another, with strong support.

Many of the phylogenetic relationships within clades K.1 and K.2 are assessed here for the first time. Phylogenetic positions of species of proposed hybrid origin, such as *Tillandsia maya* (*T. balbisiana* × *T. brachycaulos*, Ramírez and Carnevali, 2003), *T. may-patii* (*T. brachycaulos* × *T. dasyliriifolia*, Ramírez and Carnevali, 2003), and *T. rothii* (*T. jaliscomonticola* × *T. rolandgosselinii*, Rauh, 1976), were consistently recovered as sister lineages of one of their proposed parental lineages, in agreement with the proposed hybrid origin of these species. Future studies using nuclear sequence data and phylogenetic network analyses could explicitly test these hybrid origin hypotheses and explore the prevalence of hybridization across the expanded clade K and its influence in the diversification of the group.

### Time of origin and diversification of the expanded clade K

We present a time-calibrated phylogenetic framework for the expanded clade K based on a comprehensive amount of plastome sequence data. To include key nodes for secondary calibrations, a representative sampling (16 out of 22 genera) of other Tillandsioideae lineages was incorporated, allowing to date the age of the reconstructed ancestors of the expanded clade K all the way back to the stem node of Bromeliaceae. The following discussion will focus on the nodes preceding this focal group.

Regardless of the differences in phylogenetic dating method, taxonomic sampling, and calibration sources (fossils vs. secondary calibrations), most of our crown age estimates fall within the age ranges estimated in previous studies, including the crown ages of Bromeliaceae (28.69 vs. 96–19 Mya; Givnish et al., 2004; Janssen and Bremer, 2004; Givnish et al., 2007; Givnish et al., 2011; Bouchenak-Khelladi et al., 2014; Givnish et al., 2014; Zhou et al., 2018; Ramírez-Barahona et al., 2020), Core Tillandsioideae (11.42 vs. 12.9–8.7 Mya; Givnish et al., 2011; Givnish et al., 2014; Kessous et al., 2020; Loiseau et al., 2021; Möbus et al., 2021), and tribe Tillandsieae (8.8 vs. 8.8–6.5 Mya; Kessous et al., 2020; Loiseau et al., 2021; Möbus et al., 2021). In contrast, crown ages of subfamily Tillandsioideae (17.7 vs. 15.2–13.3 Mya; Givnish et al., 2011; Givnish et al., 2014; Ramírez-Barahona et al., 2020; Möbus et al., 2021) and *Tillandsia* subg. *Tillandsia* (7.34 vs. 6.6 Mya; Möbus et al., 2021) are estimated to be older than in previous studies. In both cases, differences in the employed taxon samplings and the use herein of a considerably greater amount of sequence data could explain these age differences. Our sampling, for instance, does not include representatives from tribe Glomeropitcairnieae, sampled by Givnish et al. (2011; 2014) and Ramírez-Barahona et al. (2020), which could have influenced our crown age estimates of subfamily Tillandsioideae. In the case of *T.* subg. *Tillandsia*, Möbus et al. (2021) only sampled two species, *T. utriculata* from the expanded clade K and *T. malzinei* from the *T. paniculata* clade, therefore representing the node succeeding *T.* subg. *Tillandsia* from our phylogeny for which we estimated an age of 6.94 Mya, which is closer to their age estimate for *T.* subg. *Tillandsia*. Our taxon sampling design, in combination with a comprehensive amount of sequence data, allowed us to calibrate for the first time several nodes, not only within our focal group, the expanded clade K, but also in other Tillandsioideae lineages. We expect that this dated phylogenetic framework will facilitate future macroevolutionary studies and provide reference age estimates for performing secondary calibrations for other Tillandsioideae lineages.

### Evolution in time and space of the expanded clade K

#### Origin of the expanded clade K

Our aim was to trace the geographic origin of the focal group expanded clade K, and we found the combined area of the Boreal and South Brazilian dominions (B), South American transition zone (C), and Pacific dominion (D) as the most probable ancestral area for the nodes subtending subfamily Tillandsioideae, Core Tillandsioideae, tribe Tillandsieae, and a clade including all tribe Tillandsieae species, excepting the genus *Guzmania*. The three remaining preceding nodes of the expanded clade K, including the one corresponding to *Tillandsia* subg. *Tillandsia*, were reconstructed in a smaller ancestral combined area restricted to the Boreal and South Brazilian dominions (B) and South American transition zone (C). To our knowledge, few studies have performed ancestral area reconstructions for these relevant nodes, focusing on different lineages and taxonomic scales within Bromeliaceae (Givnish et al., 2011; Pinzón et al., 2016; Granados Mendoza et al., 2017; Kessous et al., 2020). In their study of the family Bromeliaceae, Givnish et al. (2011) estimated the area of the Andes and south Chile as the most probable ancestral area for the node subtending subfamily Tillandsioideae. The latter area roughly corresponds to our area delimitation of the South American transition zone (C) and Pacific dominion (D), except for the North East portion of South America that Givnish et al. (2011) circumscribed as part of a different area that also included the Caribbean and southeastern USA, recovered as the second most probable ancestral area for this node by these authors. However, our estimation for this node needs to be taken with caution, since our taxon sampling lacks representatives of tribe Glomeropitcairnieae, which is restricted to the Lesser Antilles and northeastern Venezuela and, together with tribe Catopsideae, forms the sister lineage (non-Core Tillandsioideae) of the remaining Tillandsioideae species (Core Tillandsioideae). Therefore, this is a key lineage for inferring where subfamily Tillandsioideae arose.

The area of the ancestor of Core Tillandsioideae has been previously reconstructed in either the individual area of the Andes and southern Chile (Givnish et al., 2011) or the combined area of the Andes and southern Chile, Chacoan dominion, and Atlantic Forest (Kessous et al., 2020). Our results for this node are similar to those obtained by Givnish et al. (2011), with the exception that we recovered a combined ancestral area that also includes the Boreal and South Brazilian dominions (B). The reconstruction of the Atlantic Forest and Chacoan dominion as part of the most probable area for Core Tillandsioideae by Kessous et al. (2020) could be explained by the emphasis that these authors put on sampling Tillandsioideae representatives from these two regions. Both tribe Tillandsieae (Givnish et al., 2011; Granados Mendoza et al., 2017; Kessous et al., 2020) and *Tillandsia* subg. *Tillandsia* (Pinzón et al., 2016; Granados Mendoza et al., 2017) have been reconstructed in previous studies in areas overlapping the herein circumscribed South American transition zone (C) and Pacific dominion (D). The latter is in partial agreement with our results since, for tribe Tillandsieae, we recovered a combined area that also includes the Boreal and South Brazilian dominions (B), whereas for *T.* subg. *Tillandsia*, we reconstructed the combined area of the Boreal and South Brazilian dominions (B) and the South American transition zone (C).

Differences in the estimated ancestral areas for the nodes preceding the expanded clade K among our and previous studies are most probably the result of the alternative area circumscriptions and reconstruction strategies used across studies. Additionally, both previous studies and ours have different focal groups, which could result in geographically biased taxon samplings, impacting the obtained ancestral area probabilities at deeper nodes. The recovery of the Boreal and South Brazilian dominions (B) as part of the most probable combined area for all nodes preceding the expanded clade K in our study could be related to our denser representation of species from this area (46 spp.) compared with previous studies (1 sp., Givnish et al., 2011; 28 spp., Pinzón et al., 2016; 93 spp., Granados Mendoza et al., 2017; Kessous et al., 2020). Furthermore, various studies have documented an active interchange between the Amazonia (≈ Boreal and South Brazilian dominions) and the Andes (≈ South American transition zone; Pérez-Escobar et al., 2022), which, although not formally tested herein, could have occurred in early Tillandsioideae lineages, maintaining to a certain extent these two areas as a single unit.

Our taxonomic sample includes 17 species of *Tillandsia* subg. *Tillandsia* distributed in the Pacific dominion (D). Nonetheless, this individual area or combined areas including it were not recovered as the most probable ancestral area for this subgenus, as well as one preceding and one succeeding node, suggesting an area shift from the combined area of the Boreal and South Brazilian dominions (B) and the South American transition zone (C) to the non-contiguous individual area of the Mexican transition zone + Mesoamerican dominion + Nearctic region (F) at the node subtending the expanded clade K. Our temporal framework suggests that this non-contiguous area shift occurred at least 4.86 Mya, well after the first (12 Mya) but before the last intense building pulse of the north-central Andes (4.5 Mya; Rull and Carnaval, 2020), when the mean elevation of Central and Venezuelan Andes had already reached ca. 4,000 m and the Northern Andes ca. 3,500 m (Hoorn et al., 2010). The timing of the formation of the Panama Isthmus has been intensely debated, with studies proposing the presence of land bridges as early as 23 Mya (Bacon et al., 2015) and land closure dating estimates as recent as 2.8 Mya (O’Dea et al., 2016). Regardless of whether a land or mountain chain connection was already established by the time the ancestor of the expanded clade K dispersed from South to North and Central America, our analysis failed to identify the Pacific dominion (D), which includes the region previously occupied by the Panama Isthmus and the northern and Venezuelan Andes, for the nodes immediately preceding the expanded clade K, suggesting long-distance dispersal as a plausible explanation. Three additional, independent colonization events from the combined area of the Boreal and South Brazilian dominions (B) and the South American transition zone (C) to Central and North America are herein reconstructed for lineages within the *T. biflora* complex (clade N) and *T*. subg. *Viridantha*, as well as the genus *Pseudalcantarea*, at 3.66, 2.29, and 1.79 Mya, respectively (**Figure 3** and **Supplementary material 4**). These results suggest that long-distance dispersal could have occurred several times in the evolution of Tillandsioideae. However, the different modes of dispersal of the latter lineages should be formally assessed in future studies including denser taxon samplings for these focal groups and their close relatives.

Our biogeographical model selection favored a founder–event speciation, in support of a hypothesis of North and Central America having been colonized, if not entirely, at least by some long-distance dispersal events from larger South American tillandsioid ancestral populations, which could have been facilitated by the characteristic hair-like appendages (plumose coma) of their anemochorous seeds (Smith and Downs, 1977; Barfuss et al., 2016). *Tillandsia* seeds have small sizes, large comas, and seed coats composed of dead air-filled cells (Chilpa-Galván et al., 2018), attributes that are thought to aid in air flotation (Madison, 1977). Furthermore, compared with other anemochorous species, the terminal velocity of falling seeds in still air is slow in *Tillandsia* (Chilpa-Galván et al., 2018), characteristic of seeds with high dispersal ability, being more prone to be dispersed over long distances (Nathan et al., 2011). Long-distance dispersal from South to North and Central America has been suggested for other non-Tillandsioideae bromeliad lineages including the genus *Hechtia* (Hechtioideae), from the Andes and southern Chile at least 10.3 Mya, a species of *Aechmea* (Bromelioideae), from the Brazilian Shield ca. 5 Mya, and the genus *Fosterella* (Pitcairnioideae), from the Central Andes (Givnish et al., 2011). Additionally, Bromeliaceae are known to have undergone a transcontinental long-distance dispersal with the colonization of western Africa by *Pitcairnia feliciana* (Pitcairnioideae) from South America ca 9.3 Mya (Givnish et al., 2004). Similar long dispersal events to North and Central America have been reported in other angiosperm lineages possessing wind-dispersed seeds, such as the epiphytic genus *Cycnoches* (Orchidaceae; Pérez-Escobar et al., 2017) and the lianescent genus *Amphilophium* (Bignoniaceae; 11.2 Mya; Thode et al., 2019) from the Amazonia and the Atlantic Forest, respectively.

#### Dispersal route of the expanded clade K in the Neotropics

Within North and Central America, a region spanning the Mesoamerican dominion, herein Pacific Lowlands and Balsas Basin provinces + El Cabo de Baja California district (G) and the Mosquito, Veracruzan, and Yucatán Peninsula provinces (I), and the Mexican transition zone (H; sensu Morrone et al., 2022) is reconstructed as the ancestral area of the expanded clade K. To our knowledge, only two other studies have performed ancestral area reconstructions for this node. Granados Mendoza et al. (2017) recovered North and Central America as the most probable ancestral area for the expanded clade K, whereas Pinzón et al. (2016) reconstructed this node at an ancestral area, therein referred to as Pacific Ocean coast and mountainous region, equivalent to the Mesoamerican dominion (sensu Morrone et al., 2022; herein areas G and I). This is in general agreement with our results; however, comparison with Granados Mendoza et al. (2017) within the expanded clade K is limited by the broad area circumscription and low phylogenetic resolution of their study. Failure to reconstruct the Mosquito, Veracruzan, and Yucatán Peninsula provinces (I) as part of the ancestral area of the expanded clade K by Pinzón et al. (2016) could be attributed to differences in taxon sampling designs, which in their case was strongly focused on sampling representatives from their focal group, the *Tillandsia utriculata* species complex, which is one of the various lineages of clade K.2. Regardless of the taxon sampling differences, reconstruction for the shared sampled nodes between Pinzon et al. (2016) and the present study coincide in general.

The ancestor of the expanded clade K is herein dated to have colonized the Mesoamerican dominion and the Mexican transition zone by 4.86 Mya, a time when most of the Mexican highlands were already formed (Sierra Madre Oriental, 60–40 Mya; Sierra Madre Occidental, 54–6 Mya; Sierra Madre del Sur, 60–18 Mya; Mastretta-Yanes et al., 2015). Models suggest that during the Pliocene (5.33–2.58 Mya) these highlands were mostly dominated by tropical semi-deciduous and deciduous forest and warm-temperate mixed forest in their central to southernmost portions, and temperate conifer forest in the northernmost portion of the Sierra Madre Oriental (Salzmann et al., 2011). Among these vegetation types, the conifer forest, which together with the tropical–subtropical dry and moist broadleaf forests concentrate most of the expanded clade K species diversity, is modeled to have expanded to the Sierra Madre Occidental toward the present, following the global mean temperature colling trend (Salzmann et al., 2011; Mastretta-Yanes et al., 2015). The two major lineages of the expanded clade K, clades K.1 and K.2, began diversifying ca. 1 to 2 My later within the same ancestral area. Then, a series of distribution range expansions and contractions occurred within these two clades, the majority of them in a period (2.8 Mya and onward) characterized by pronounced climate fluctuations, derived from the glacial–interglacial climate oscillations (2.58 Mya to 11.7 Kya; Cohen and Gibbard, 2011), and pronounced volcanic activity, mainly in the Trans-Mexican Volcanic Belt (1.5 Mya to date; revised by Mastretta-Yanes et al., 2015). Pleistocene temperature oscillations are thought to have generated changes in precipitation and seasonality, promoting highland vegetation elevational shifts, deriving in cycles of reduced gene flow at high-elevation refugia (interglacial periods) and admixture at lower elevations (glacial periods; Mastretta-Yanes et al., 2015). In addition to these climatic oscillations, the volcanic activity of the Trans-Mexican Volcanic Belt during the Pleistocene, persistent until today, derived in pronounced landscape modifications with the formation of large (> 3500 m) stratovolcanoes, which could not only offer new habitats for colonization but also build new highland areas where the cycles of reduced gene flow and admixture could have taken place (Mastretta-Yanes et al., 2015). The genetic effects of such elevation shifts are expected to vary across plant lineages depending on their ecological affinities, with less cold-tolerant groups being more prone to experience gene flow at lower elevations during glacial periods (oaks, Cavender-Bares et al., 2011, and pines, Moreno-Letelier et al., 2013). Additionally, taxa particularly sensitive to changes in precipitation could have experienced relevant genetic consequences resulting from expansions and contractions of habitats with suitable humid conditions, such as those distributed in cloud forests, which are known to hold a substantial diversity of epiphytic lineages (Ramírez-Barahona and Eguiarte, 2013; Pérez-Escobar et al., 2022). The expanded clade K is composed of mostly epiphytic and epilithic species, two lifestyles particularly sensitive to water availability (Zotz and Hietz, 2001); therefore, Pleistocene precipitation oscillations could have had a significant effect in their genetic diversity and geographic distribution. Furthermore, the resulting mosaic of dry, semidry, and humid forest that characterize the Mexican highlands (Soberón Mainero, 2008) could have been associated with the evolution of photosynthetic metabolisms, as the expanded clade K is known to comprise both C3 and CAM representatives (Crayn et al., 2015; De La Harpe et al., 2020; Groot Crego et al., 2023). Additionally, as discussed in the previous section, the high dispersal capacity of the *Tillandsia* seeds could have played a significant role in their ability to colonize new areas within North and Central America and the Caribbean.

Colonization of the Antillean subregion + SE USA (E) is here reconstructed exclusively within clade K.2 in six independent events occurring not earlier than 0.68 Mya (*Tillandsia bartramii*) and as recent as 60 Kya (*T. schiedeana*). Within the Antillean subregion + SE USA (E), *T. schiedeana*, *T. capitata*, and *T. streptophylla* are only found on the Caribbean islands, *T. variabilis* and *T. setacea* are distributed both on the Caribbean islands and in southeastern USA, and *T. bartramii* is restricted to southeastern USA. These six species display the CAM photosynthetic pathway (Crayn et al., 2015) and have ample geographic distributions, and some of them are known to form natural hybrids (*T. schiedeana*, *T. bartramii*, *T. variabilis*, and *T. streptophylla*; Rauh, 1976; Luther, 1985; Ramírez et al., 2000; Ehlers, 2004). Nine out of 10 dispersal events to the Pacific and Boreal and South Brazilian dominions + South American transition zone (Y) were similarly only reconstructed within clade K.2 from 1.77 to 0.01 Mya, except for *T. punctulata* of clade K.1, from 2.35 Mya. The latter dispersal events exclusively involved range expansions to the Pacific dominion (D), suggesting that equatorward dispersal of the expanded clade K occurred only at shallower divergences, at the earliest 2.35 Mya. Future phylogeographic studies combining population-level samplings representing the breadth of the geographic distribution of the species (as in Pinzón et al., 2016; Ancona et al., 2022), nuclear NGS-derived multilocus data, and explicit biogeographical hypotheses could permit addressing how and when these widely distributed species colonized the Caribbean region and Pacific dominion and formally testing which morpho-physiological attributes could have facilitated their dispersal.

Although older than the expanded clade K (crown age 6.65 Mya), the genus *Hechtia* (Hechtioideae) shows a similar biogeographical pattern, with most of its diversification occurring during the last 4 My, albeit specializing into arid and semiarid biomes, rather than into the ample range of climatic conditions inhabited by the species of the expanded clade K. Several taxa have been proposed to have diversified synchronously with the expanded clade K within the Mexican highlands (e.g., *Pseudotsuga menziesii*, Gugger et al., 2011; *Juniperus deppeana*, Martínez de León et al., 2022; *Quercus* series *Virentes*, Cavender-Bares et al., 2011; *Podocarpus matudae*, Ornelas et al., 2010; and *Dioon*, Dorsey et al., 2018) and, although their dispersal histories could have been shaped by the same climatic and orogenic processes, their biogeographic histories are as diverse as their phylogenetic and ecological affinities, adaptations, and geographic origins.

## Conclusion

We newly sequenced and assembled 162 Tillandsioideae species using the cost-effective sequencing strategy Hyb-Seq, considerably expanding existing bromeliad genomic resources. While some recalcitrant nodes at the backbone of Core Tillandsieae and at some shallower divergences within clade K.2 remain with low statistical support, the analysis of complete plastomes resulted in an overall highly supported phylogenetic framework. The additional phylogenetic resolution herein obtained allowed to uncover new relationships that could aid in defining which morphological attributes characterize previously delimited polymorphic species groups, such as the *Tillandsia biflora* complex. Our phylogenetic context points toward the need for further revision of the currently adopted classification in the light of additional sequence data and considering evolutionary processes, such as hybridization, to interpret the consistently recovered phylogenomic discordance between nuclear and plastid evidence. The expanded clade K colonized the Mexican transition zone and Mesoamerican dominion by long-distance dispersal from a combined area of the Boreal and South Brazilian dominions and South American transition zone at least 4.86 Mya. Several subsequent dispersion events occurred northward to the Nearctic, eastward to the Caribbean, and southward to the Pacific dominion during the last 2.8 Mya. The pronounced climate fluctuations, derived from the glacial–interglacial climate oscillations, and the volcanic activity of the Trans-Mexican Volcanic Belt could have played an important role in the dispersal history of the expanded clade K. Future phylogeographic studies combining population- level sampling representing the breadth of the geographic distribution of the species, nuclear NGS-derived multilocus data, and explicit biogeographical hypotheses, could address how and when widely distributed species colonized the Caribbean region and Pacific dominion and formally test which morpho-physiological attributes could have facilitated their dispersal. Finally, future studies using nuclear sequence data and phylogenetic network analyses could formally test previous hybrid origin hypotheses and explore the prevalence of hybridization across the expanded clade K, as well as other Tillandsioideae lineages, and its influence in the diversification of these groups.

## Data availability statement

The datasets presented in this study can be found in online repositories. The names of the repository/repositories and accession number(s) can be found below: Genbank, accession numbers OQ935587 - OQ935748.

## Author contributions

SV-P and CGM conceived and designed the study. SV-P, CGM, DD, AR, CM-A, GS, EG, IR-M, MF-C, XG-A, MB, AM-G, CH-L, MB, and SW designed the taxon sampling and collected or provided the samples. SV-P, CGM, DD, LC, and GS performed the laboratory work. SV-P, DD, MJ, AR, CM-A, and LC performed the bioinformatic process. SV-P, CGM, CM-A, RH-G, SM, and LS-G designed and performed the analyses. SV-P drafted the manuscript. SV-P, CGM, DD, MJ, AR, CM-A, RH-G, SM, LS-G, GS, EG, IR-M, LC, MF-C, XG-A, AM-G, CH-L, MB, and SW proofread and approved the final manuscript. All authors contributed to the article and approved the submitted version.

## Supplementary material

Supplementary material 1 Taxon sampling and voucher information including collector, and collection number (herbarium code as in http://sweetgum.nybg.org/science/ih/).

Supplementary material 2
**(A)** Sources of geographic information. **(B)** Species’ area coding used in the analysis of the complete taxon sampling. **(C)** Species’ area coding used in the analysis of *Tillandsia* subg. *Tillandsia*. **(D)** Node probabilities resulting from the analysis of the complete taxon sampling. **(E)** Node probabilities resulting from the analysis of *T*. subg. *Tillandsia*.

Supplementary material 3
**(A)** Reference tree with nodes numbered as cited in **Supplementary material 2D**, corresponding to the analysis of the complete taxon sampling. **(B)** Reference tree with nodes numbered as cited in **Supplementary material 2E**, corresponding to the analysis of *Tillandsia* subg. *Tillandsia*. **(C)** Modification of **Figure 6** showing the first and second most probable individual or combined ancestral areas recovered under the BAYAREALIKE+J model for highly supported (PP ≥ 0.85) internal nodes. **(D)** Modification of **Figure 7** showing the first and second most probable individual or combined ancestral areas recovered under the BAYAREALIKE+J model for highly supported (PP ≥ 0.85) internal nodes.

Supplementary material 4 Recovered stem and crown ages for main lineages discussed in the text along with their minimum and maximum 95% HPD intervals.

Supplementary material 5 Phylogenetic relationships obtained from an IQ-TREE maximum likelihood analysis of the complete taxon sampling to illustrate a long branch (blue arrow) subtending *Tillandsia disticha*. Two strongly supported phylogenetic incongruencies relative to the BEAST2 analysis are denoted with red arrows.
