# Supplementary material for: Plastome phylogenomics reveals an early Pliocene North- and Central America colonization by long-distance dispersal from South America of a highly diverse bromeliad lineage
Source: Front Plant Sci. 2023 Jun 23;14:1205511. doi: 10.3389/fpls.2023.1205511 (PMC10326849; doi:10.3389/fpls.2023.1205511)
Supplement: Supplementary material 1 — Taxon sampling and voucher information including collector, and collection number (herbarium code as in http://sweetgum.nybg.org/science/ih/). [file Image_1.pdf]

A

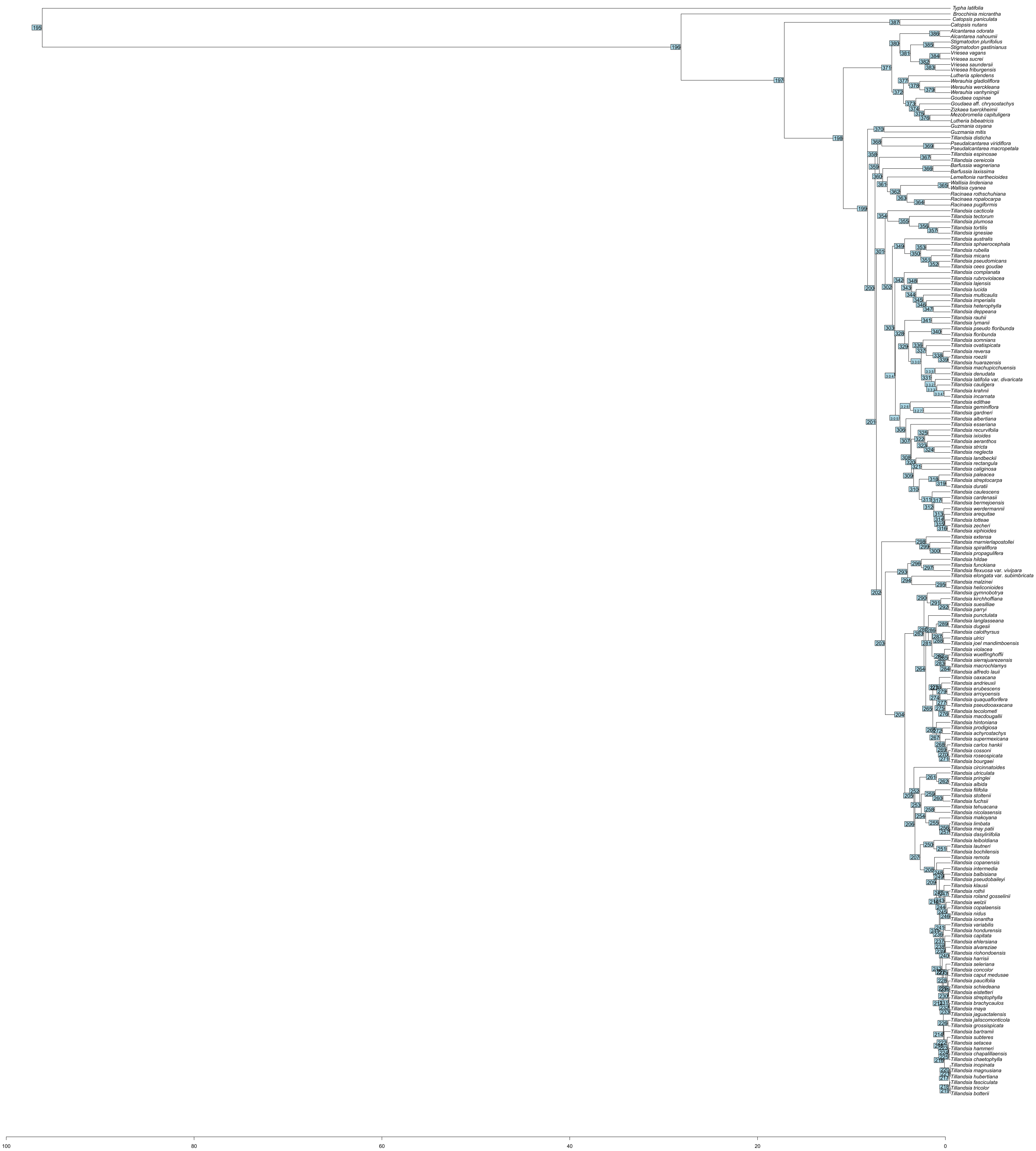

Typha latifolia
Brocchinia micrantha
Catopsis paniculata
Catopsis nutans
Alcantarea odorata
Alcantarea nahoumii
Stigmatodon plurifolius
Stigmatodon gastinianus
Vriesea vagans
Vriesea sucrei
Vriesea saundersii
Vriesea friburgensis
Lutheria splendens
Werauhia gladioliflora
Werauhia werckleana
Werauhia vanhyningii
Goudaea ospinae
Goudaea aff. chrysostachys
Zizkaea tuerckheimii
Mezobromelia capituligera
Lutheria bibeatricis
Guzmania osyana
Guzmania mitis
Tillandsia disticha
Pseudalcantarea viridiflora
Pseudalcantarea macropetala
Tillandsia espinosae
Tillandsia cereicola
Barfussia wagneriana
Barfussia laxissima
Lemeltonia narthecioides
Wallisia lindeniana
Wallisia cyanea
Racinaea rothschuhiana
Racinaea ropalocarpa
Racinaea pugiformis
Tillandsia cacticola
Tillandsia tectorum
Tillandsia plumosa
Tillandsia tortilis
Tillandsia ignesiae
Tillandsia australis
Tillandsia sphaerocephala
Tillandsia rubella
Tillandsia micans
Tillandsia pseudomicans
Tillandsia cees goudae
Tillandsia complanata
Tillandsia rubroviolacea
Tillandsia lajensis
Tillandsia lucida
Tillandsia multicaulis
Tillandsia imperialis
Tillandsia heterophylla
Tillandsia deppeana
Tillandsia rauhii
Tillandsia lymanii
Tillandsia pseudo floribunda
Tillandsia floribunda
Tillandsia somnians
Tillandsia ovatispicata
Tillandsia reversa
Tillandsia roezlii
Tillandsia huarazensis
Tillandsia machupicchuensis
Tillandsia denudata
Tillandsia latifolia var. divaricata
Tillandsia cauligera
Tillandsia krahnii
Tillandsia incarnata
Tillandsia edithae
Tillandsia geminiflora
Tillandsia gardneri
Tillandsia albertiana
Tillandsia esseriana
Tillandsia recurvifolia
Tillandsia ixioides
Tillandsia aeranthos
Tillandsia stricta
Tillandsia neglecta
Tillandsia landbeckii
Tillandsia rectangula
Tillandsia caliginosa
Tillandsia paleacea
Tillandsia streptocarpa
Tillandsia duratii
Tillandsia caulescens
Tillandsia cardenasii
Tillandsia bermejoensis
Tillandsia werdermannii
Tillandsia arequitae
Tillandsia lotteae
Tillandsia zecheri
Tillandsia xiphioides
Tillandsia extensa
Tillandsia marnierlapostollei
Tillandsia spiraliflora
Tillandsia propagulifera
Tillandsia hildae
Tillandsia funckiana
Tillandsia flexuosa var. vivipara
Tillandsia elongata var. subimbricata
Tillandsia malzinei
Tillandsia heliconioides
Tillandsia gymnobotrya
Tillandsia kirchhoffiana
Tillandsia suesilliae
Tillandsia parryi
Tillandsia punctulata
Tillandsia langlasseana
Tillandsia dugesii
Tillandsia calothyrsus
Tillandsia ulrici
Tillandsia joel mandimboensis
Tillandsia violacea
Tillandsia wuelfinghoffii
Tillandsia sierrajuarezensis
Tillandsia macrochlamys
Tillandsia alfredo lauii
Tillandsia oaxacana
Tillandsia andrieuxii
Tillandsia erubescens
Tillandsia arroyoensis
Tillandsia quaquaflorifera
Tillandsia pseudooaxacana
Tillandsia tecolometl
Tillandsia macdougallii
Tillandsia hintoniana
Tillandsia prodigiosa
Tillandsia achyrostachys
Tillandsia supermexicana
Tillandsia carlos hankii
Tillandsia cossoni
Tillandsia roseospicata
Tillandsia bourgaei
Tillandsia circinnatoides
Tillandsia utriculata
Tillandsia pringlei
Tillandsia albida
Tillandsia filifolia
Tillandsia stoltenii
Tillandsia fuchsii
Tillandsia tehuacana
Tillandsia nicolasensis
Tillandsia makoyana
Tillandsia limbata
Tillandsia may patii
Tillandsia dasyliriifolia
Tillandsia leiboldiana
Tillandsia lautneri
Tillandsia bochilensis
Tillandsia remota
Tillandsia copanensis
Tillandsia intermedia
Tillandsia balbisiana
Tillandsia pseudobaileyi
Tillandsia klausii
Tillandsia rothii
Tillandsia roland gosselinii
Tillandsia welzii
Tillandsia copalaensis
Tillandsia nidus
Tillandsia ionantha
Tillandsia variabilis
Tillandsia hondurensis
Tillandsia capitata
Tillandsia ehlersiana
Tillandsia alvareziae
Tillandsia riohondoensis
Tillandsia harrisii
Tillandsia seleriana
Tillandsia concolor
Tillandsia caput medusae
Tillandsia paucifolia
Tillandsia schiedeana
Tillandsia eistetteri
Tillandsia streptophylla
Tillandsia brachycaulos
Tillandsia maya
Tillandsia jaguactalensis
Tillandsia jaliscomonticola
Tillandsia grossispicata
Tillandsia bartramii
Tillandsia subteres
Tillandsia setacea
Tillandsia hammeri
Tillandsia chapalillaensis
Tillandsia chaetophylla
Tillandsia inopinata
Tillandsia magnusiana
Tillandsia hubertiana
Tillandsia fasciculata
Tillandsia tricolor
Tillandsia botterii
100
80
60
40
20
0

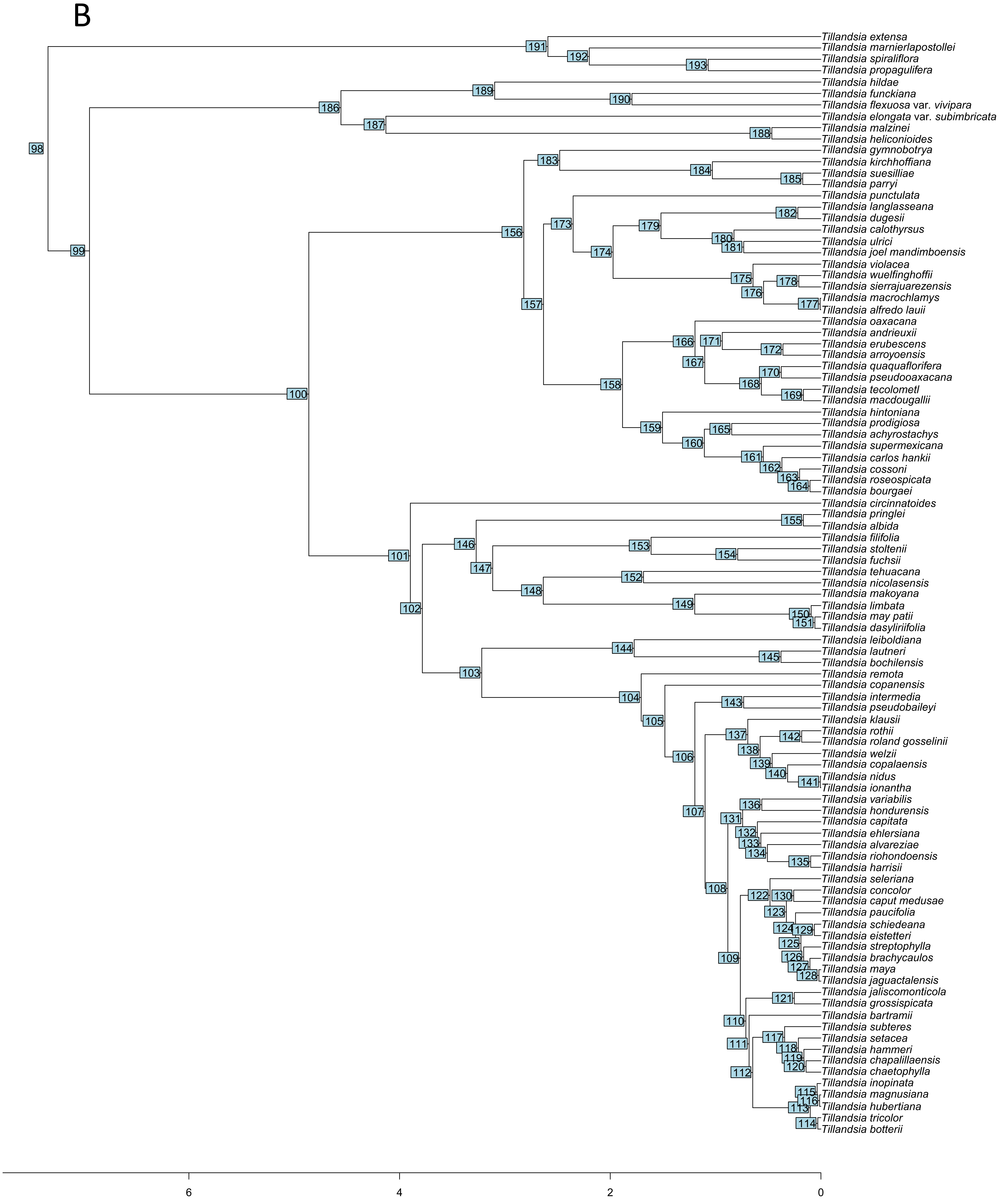

B
Tillandsia extensa
Tillandsia marnierlapostollei
Tillandsia spiraliflora
Tillandsia propagulifera
Tillandsia hildae
Tillandsia funckiana
Tillandsia flexuosa var. vivipara
Tillandsia elongata var. subimbricata
Tillandsia malzinei
Tillandsia heliconioides
Tillandsia gymnobotrya
Tillandsia kirchhoffiana
Tillandsia suesilliae
Tillandsia parryi
Tillandsia punctulata
Tillandsia langlasseana
Tillandsia dugesii
Tillandsia calothyrsus
Tillandsia ulrici
Tillandsia joel mandimboensis
Tillandsia violacea
Tillandsia wuelfinghoffii
Tillandsia sierrajuarezensis
Tillandsia macrochlamys
Tillandsia alfredo lauii
Tillandsia oaxacana
Tillandsia andrieuxii
Tillandsia erubescens
Tillandsia arroyoensis
Tillandsia quaquaflorifera
Tillandsia pseudooaxacana
Tillandsia tecolometl
Tillandsia macdougallii
Tillandsia hintoniana
Tillandsia prodigiosa
Tillandsia achyrostachys
Tillandsia supermexicana
Tillandsia carlos hankii
Tillandsia cossoni
Tillandsia roseospicata
Tillandsia bourgaei
Tillandsia circinnatoides
Tillandsia pringlei
Tillandsia albida
Tillandsia filifolia
Tillandsia stoltenii
Tillandsia fuchsii
Tillandsia tehuacana
Tillandsia nicolasensis
Tillandsia makoyana
Tillandsia limbata
Tillandsia may patii
Tillandsia dasyliriifolia
Tillandsia leiboldiana
Tillandsia lautneri
Tillandsia bochilensis
Tillandsia remota
Tillandsia copanensis
Tillandsia intermedia
Tillandsia pseudobaileyi
Tillandsia klausii
Tillandsia rothii
Tillandsia roland gosselinii
Tillandsia welzii
Tillandsia copalaensis
Tillandsia nidus
Tillandsia ionantha
Tillandsia variabilis
Tillandsia hondurensis
Tillandsia capitata
Tillandsia ehlersiana
Tillandsia alvareziae
Tillandsia riohondoensis
Tillandsia harrisii
Tillandsia seleriana
Tillandsia concolor
Tillandsia caput medusae
Tillandsia paucifolia
Tillandsia schiedeana
Tillandsia eistetteri
Tillandsia streptophylla
Tillandsia brachycaulos
Tillandsia maya
Tillandsia jaguactalensis
Tillandsia jaliscomonticola
Tillandsia grossispicata
Tillandsia bartramii
Tillandsia subteres
Tillandsia setacea
Tillandsia hammeri
Tillandsia chapalillaensis
Tillandsia chaetophylla
Tillandsia inopinata
Tillandsia magnusiana
Tillandsia hubertiana
Tillandsia tricolor
Tillandsia botterii
6
4
2
0

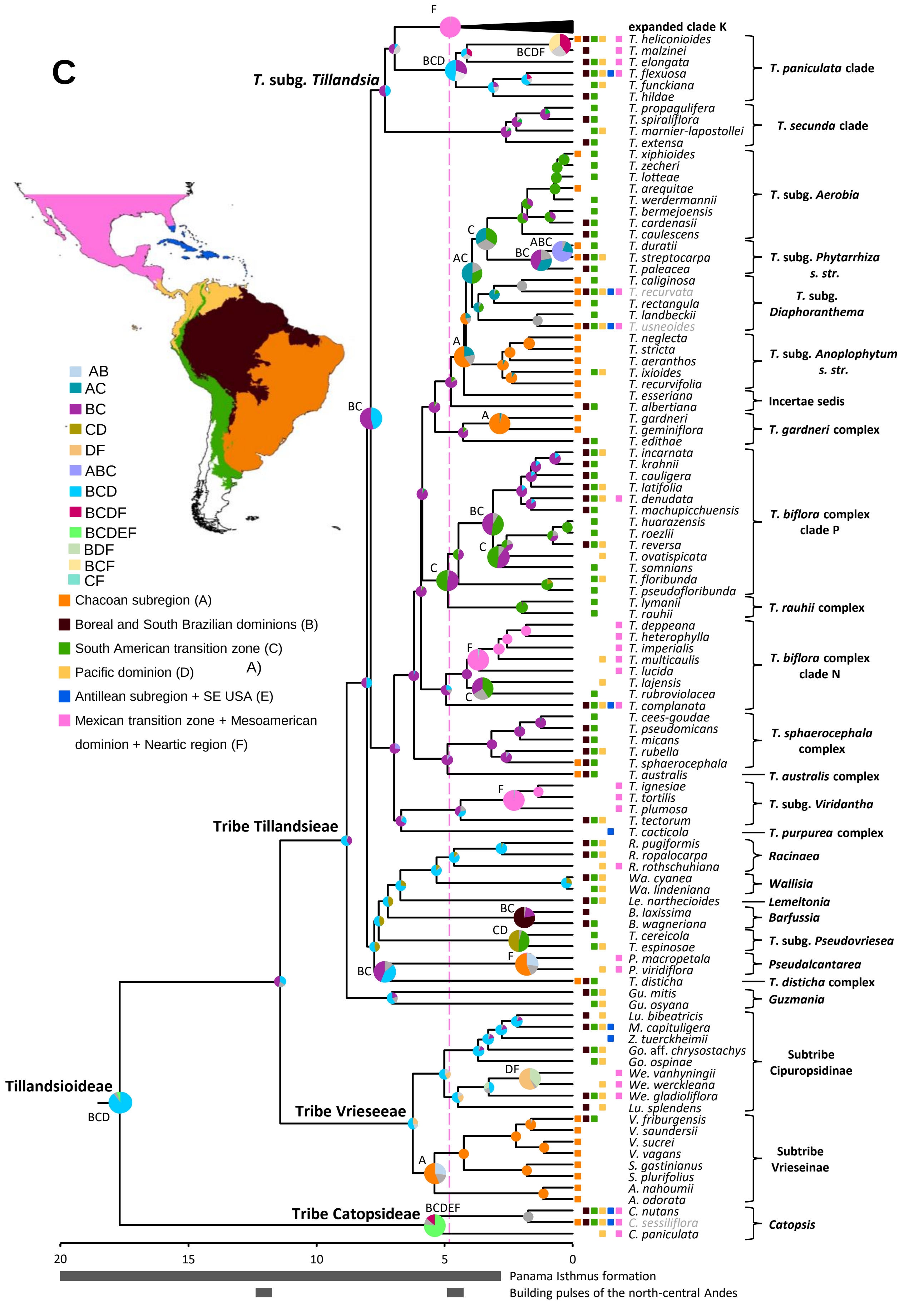

C
expanded clade K
T. heliconioides
T. malzinei
T. elongata
T. flexuosa
T. funckiana
T. hildae
T. paniculata clade
T. propagulifera
T. spiraliflora
T. marnier-lapostollei
T. extensa
T. secunda clade
T. xiphioides
T. zecheri
T. lotteae
T. arequitae
T. werdermannii
T. bermejoensis
T. cardenasii
T. caulescens
T. subg. Aerobia
T. duratii
T. streptocarpa
T. paleacea
T. subg. Phytarrhiza s. str.
T. caliginosa
T. recurvata
T. rectangula
T. landbeckii
T. usneoides
T. subg. Diaphoranthema
T. neglecta
T. stricta
T. aeranthos
T. ixioides
T. recurvifolia
T. subg. Anoplophytum s. str.
T. esseriana
Incertae sedis
T. albertiana
T. gardneri
T. geminiflora
T. edithae
T. gardneri complex
T. incarnata
T. krahnii
T. cauligera
T. latifolia
T. denudata
T. machupicchuensis
T. huarazensis
T. roezlii
T. reversa
T. ovatispicata
T. somnians
T. floribunda
T. pseudofloribunda
T. biflora complex clade P
T. lymanii
T. rauhii
T. rauhii complex
T. deppeana
T. heterophylla
T. imperialis
T. multicaulis
T. lucida
T. lajensis
T. rubroviolacea
T. complanata
T. biflora complex clade N
T. cees-goudae
T. pseudomicans
T. micans
T. rubella
T. sphaerocephala
T. sphaerocephala complex
T. australis
T. australis complex
T. ignesiae
T. tortilis
T. plumosa
T. tectorum
T. subg. Viridantha
T. cacticola
T. purpurea complex
R. pugiformis
R. ropalocarpa
R. rothschuhiana
Racinaea
Wa. cyanea
Wa. lindeniana
Wallisia
Le. narthecioides
Lemeltonia
B. laxissima
B. wagneriana
Barfussia
T. cereicola
T. espinosae
T. subg. Pseudovriesea
P. macropetala
P. viridiflora
Pseudalcantarea
T. disticha
T. disticha complex
Gu. mitis
Gu. osyana
Guzmania
Lu. bibeatricis
M. capituligera
Z. tuerckheimii
Go. aff. chrysostachys
Go. ospinae
We. vanhyningii
We. werckleana
We. gladioliflora
Lu. splendens
Subtribe Cipuropsidinae
V. friburgensis
V. saundersii
V. sucrei
V. vagans
S. gastinianus
S. plurifolius
A. nahoumii
A. odorata
Subtribe Vrieseinae
C. nutans
C. sessiliflora
C. paniculata
Catopsis
T. subg. Tillandsia
Tribe Tillandsieae
Tribe Vrieseeae
Tribe Catopsideae
Tillandsioideae
F
BCD
BCDF
C
AC
ABC
BC
A
CD
DF
BCDEF
AB
AC
BC
CD
DF
ABC
BCD
BCDF
BCDEF
BDF
BCF
CF
Chacoan subregion (A)
Boreal and South Brazilian dominions (B)
South American transition zone (C)
Pacific dominion (D)
Antillean subregion + SE USA (E)
Mexican transition zone + Mesoamerican dominion + Neartic region (F)
A)
20
15
10
5
0
Panama Isthmus formation
Building pulses of the north-central Andes

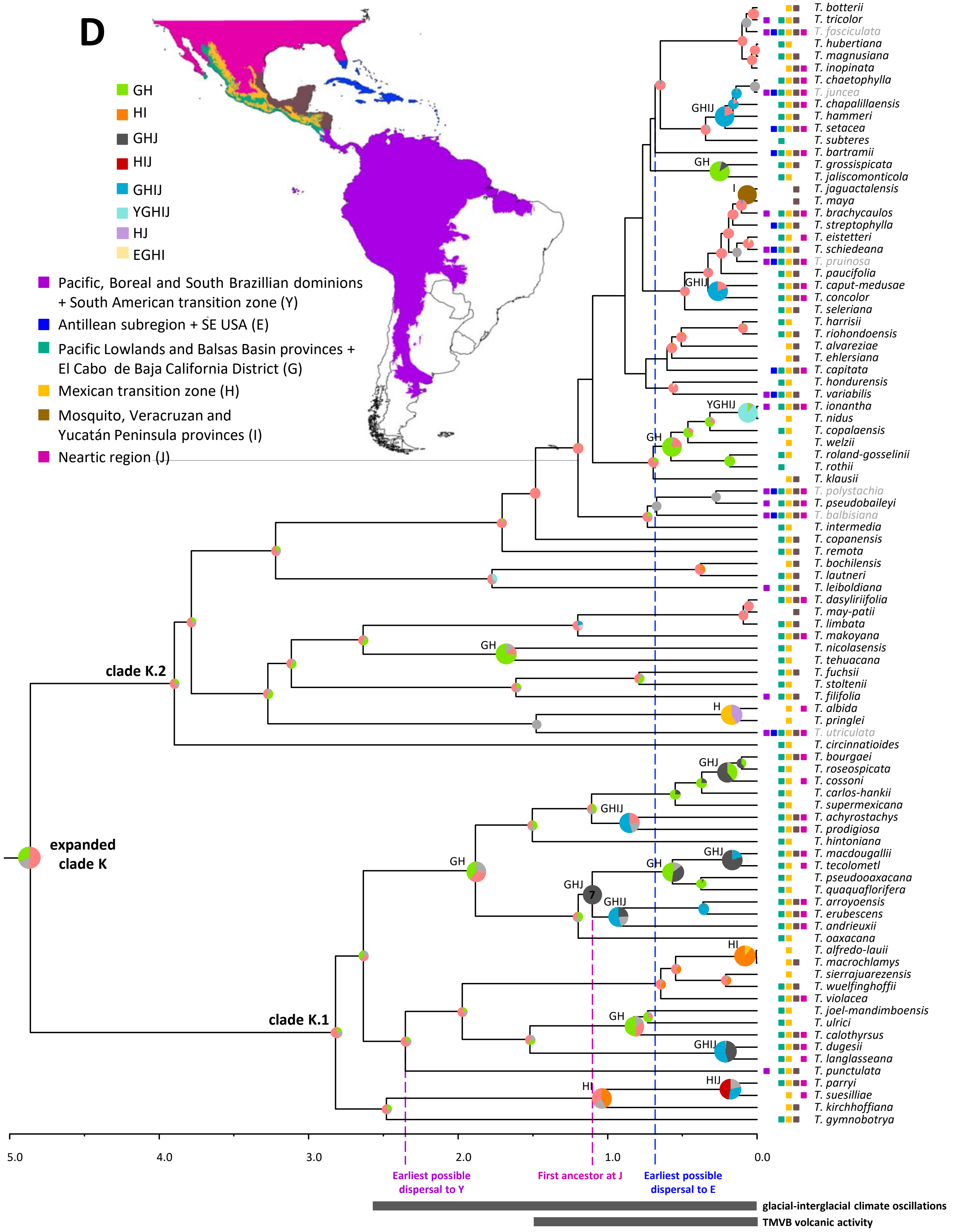

D
GH
HI
GHJ
HIJ
GHIJ
YGHIJ
HJ
EGHI
Pacific, Boreal and South Brazillian dominions + South American transition zone (Y)
Antillean subregion + SE USA (E)
Pacific Lowlands and Balsas Basin provinces + El Cabo de Baja California District (G)
Mexican transition zone (H)
Mosquito, Veracruzan and Yucatán Peninsula provinces (I)
Neartic region (J)
expanded clade K
clade K.2
clade K.1
T. botterii
T. tricolor
T. fasciculata
T. hubertiana
T. magnusiana
T. inopinata
T. chaetophylla
T. juncea
T. chapalillaensis
T. hammeri
T. setacea
T. subteres
T. bartramii
T. grossispicata
T. jaliscomonticola
T. jaguactalensis
T. maya
T. brachycaulos
T. streptophylla
T. eistetteri
T. schiedeana
T. pruinosa
T. paucifolia
T. caput-medusae
T. concolor
T. seleriana
T. harrisii
T. riohondoensis
T. alvareziae
T. ehlersiana
T. capitata
T. hondurensis
T. variabilis
T. ionantha
T. nidus
T. copalaensis
T. welzii
T. roland-gosselinii
T. rothii
T. klausii
T. polystachia
T. pseudobaileyi
T. balbisiana
T. intermedia
T. copanensis
T. remota
T. bochilensis
T. lautneri
T. leiboldiana
T. dasyliriifolia
T. may-patii
T. limbata
T. makoyana
T. nicolasensis
T. tehuacana
T. fuchsii
T. stoltenii
T. filifolia
T. albida
T. pringlei
T. utriculata
T. circinnatioides
T. bourgaei
T. roseospicata
T. cossoni
T. carlos-hankii
T. supermexicana
T. achyrostachys
T. prodigiosa
T. hintoniana
T. macdougallii
T. tecolometl
T. pseudooaxacana
T. quaquaflorifera
T. arroyoensis
T. erubescens
T. andrieuxii
T. oaxacana
T. alfredo-lauii
T. macrochlamys
T. sierrajuarezensis
T. wuelfinghoffii
T. violacea
T. joel-mandimboensis
T. ulrici
T. calothyrsus
T. dugesii
T. langlasseana
T. punctulata
T. parryi
T. suesilliae
T. kirchhoffiana
T. gymnobotrya
5.0
4.0
3.0
2.0
1.0
0.0
Earliest possible dispersal to Y
First ancestor at J
Earliest possible dispersal to E
glacial-interglacial climate oscillations
TMVB volcanic activity
