# Supplementary figures and images for: Plastome phylogenomics reveals an early Pliocene North- and Central America colonization by long-distance dispersal from South America of a highly diverse bromeliad lineage
Source: Front Plant Sci. 2023 Jun 23;14:1205511. doi: 10.3389/fpls.2023.1205511 (PMC10326849; doi:10.3389/fpls.2023.1205511)

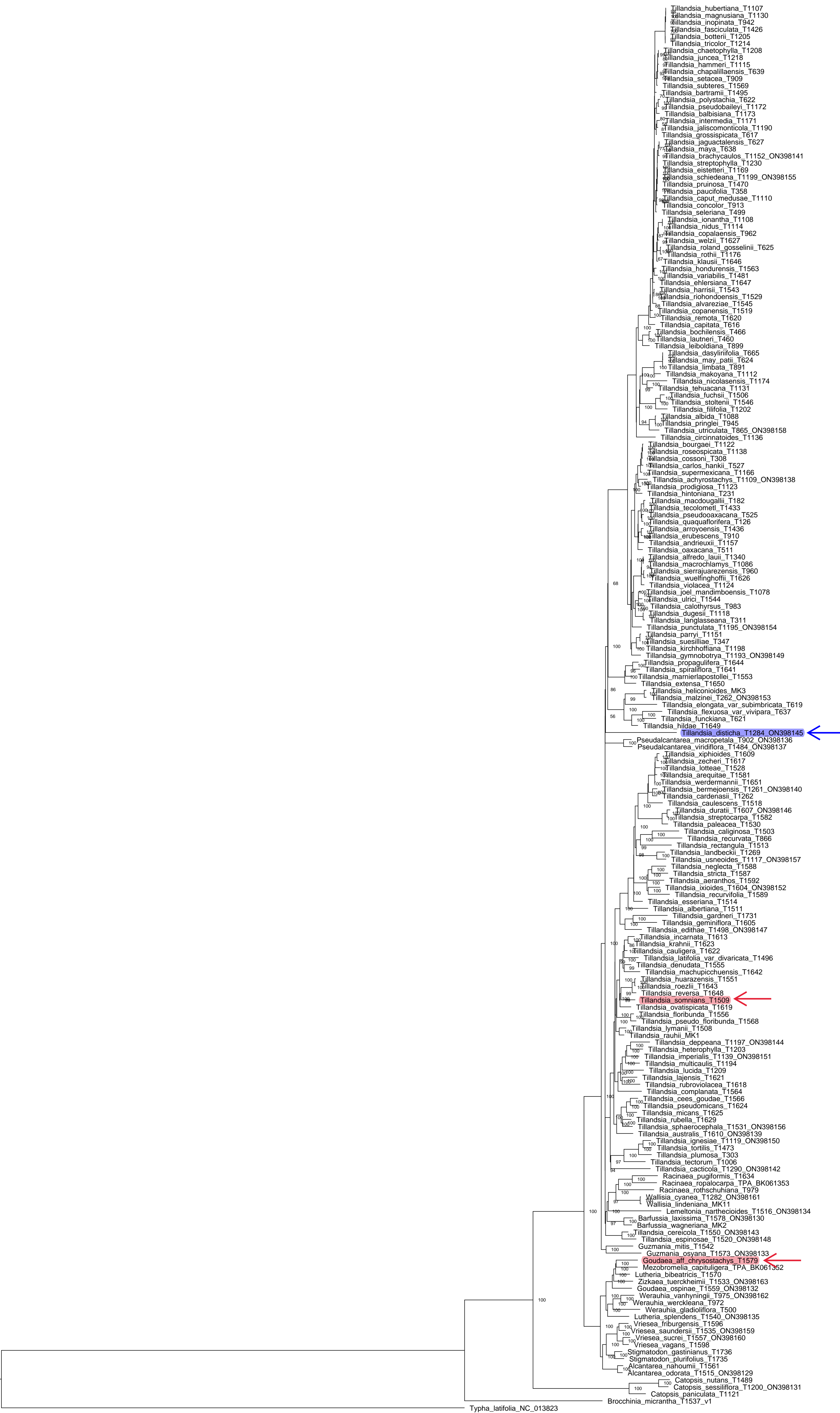

Supplement: Supplementary material 2 — (A) Sources of geographic information. (B) Species’ area coding used in the analysis of the complete taxon sampling. (C) Species’ area coding used in the analysis of Tillandsia subg. Tillandsia. (D) Node probabilities resulting from the analysis of the complete taxon sampling. (E) Node probabilities resulting from the analysis of T. subg. Tillandsia. [file Image_2.pdf]
